# Freshwater viral metagenome reveals novel and functional phage-borne antibiotic resistance genes

**DOI:** 10.1186/s40168-020-00863-4

**Published:** 2020-06-01

**Authors:** Kira Moon, Jeong Ho Jeon, Ilnam Kang, Kwang Seung Park, Kihyun Lee, Chang-Jun Cha, Sang Hee Lee, Jang-Cheon Cho

**Affiliations:** 1 Department of Biological Sciences, Inha University, Incheon, 22212 Republic of Korea; 2 National Leading Research Laboratory of Drug Resistance Proteomics, Department of Biological Sciences, Myongji University, 116 Myongjiro, Yongin, Gyeonggi-do 17058 Republic of Korea; 3 Department of Systems Biotechnology and Center for Antibiotic Resistome, Chung-Ang University, Anseong, Gyeonggi-do 17546 Republic of Korea

**Keywords:** Bacteriophage, Viral metagenome, Virome, Antibiotic resistance gene, Minimum inhibitory concentration, β-lactamase, River

## Abstract

**Background:**

Antibiotic resistance developed by bacteria is a significant threat to global health. Antibiotic resistance genes (ARGs) spread across different bacterial populations through multiple dissemination routes, including horizontal gene transfer mediated by bacteriophages. ARGs carried by bacteriophages are considered especially threatening due to their prolonged persistence in the environment, fast replication rates, and ability to infect diverse bacterial hosts. Several studies employing qPCR and viral metagenomics have shown that viral fraction and viral sequence reads in clinical and environmental samples carry many ARGs. However, only a few ARGs have been found in viral contigs assembled from metagenome reads, with most of these genes lacking effective antibiotic resistance phenotypes. Owing to the wide application of viral metagenomics, nevertheless, different classes of ARGs are being continuously found in viral metagenomes acquired from diverse environments. As such, the presence and functionality of ARGs encoded by bacteriophages remain up for debate.

**Results:**

We evaluated ARGs excavated from viral contigs recovered from urban surface water viral metagenome data. In virome reads and contigs, diverse ARGs, including polymyxin resistance genes, multidrug efflux proteins, and β-lactamases, were identified. In particular, when a lenient threshold of *e* value of ≤ 1 × e^−5^ and query coverage of ≥ 60% were employed in the Resfams database, the novel β-lactamases *bla*_HRV-1_ and *bla*_HRVM-1_ were found. These genes had unique sequences, forming distinct clades of class A and subclass B3 β-lactamases, respectively. Minimum inhibitory concentration analyses for *E. coli* strains harboring *bla*_HRV-1_ and *bla*_HRVM-1_ and catalytic kinetics of purified HRV-1 and HRVM-1 showed reduced susceptibility to penicillin, narrow- and extended-spectrum cephalosporins, and carbapenems. These genes were also found in bacterial metagenomes, indicating that they were harbored by actively infecting phages.

**Conclusion:**

Our results showed that viruses in the environment carry as-yet-unreported functional ARGs, albeit in small quantities. We thereby suggest that environmental bacteriophages could be reservoirs of widely variable, unknown ARGs that could be disseminated via virus-host interactions.

Video abstract.

## Background

Continually emerging antibiotic resistance of pathogenic bacteria is a significant threat to global health. The World Health Organization (WHO) reported that from 2017 to 2018, approximately 1.1 million patients from 69 monitoring countries were infected with antibiotic resistant pathogens [1], and this number is expected to rise. Antibiotic resistant bacteria are a compelling health issue because they make antibiotic treatments obsolete, even leading to the death of the patient. Bacteria naturally produce antimicrobial substances to disrupt neighboring competitors, and accordingly, targeted bacteria develop antibiotic resistance genes (ARGs) to defend against such mechanisms. Excessive use of antibiotics in medical practice has been implicated to accelerate development and dissemination of antibiotic resistance among bacterial populations in clinical and natural environments. ARGs can be spread from one bacterial cell to another through horizontal gene transfer (HGT) [2]. HGT of ARGs is commonly mediated by conjugation that requires physical contact between bacterial cells via pili or adhesins [3]. Conversely, transduction of ARGs mediated by bacteriophages (phages) is rare but remains a significant dissemination route of ARGs.

Although phages are the smallest and simplest biological entities on earth, they have significant ecological roles as bacterial population controllers through lytic infections and contributors to bacterial gene diversification. During infection, phages randomly incorporate bacterial genome fragments into phage capsids or genomes as they hijack the hosts’ genome replication machinery. Consequently, those genome fragments can be transferred to subsequent bacterial host genomes as phages infect other host cells. Some phages are known to have wide host range, a few with even of different bacterial orders [4], thereby functioning as a bridge of genetic exchange between different bacterial populations. Therefore, phage-associated ARGs are considered especially significant due to their possibility for wide dissemination [5, 6]. Since phages are not directly influenced by antibiotics, the presence of ARGs is best evaluated by identifying nucleic acids in phage particles. ARGs conferring resistance to lactamase [7], quinolone [8], and vancomycin [9] antibiotics were detected across diverse environments based on PCR of viral DNA. However, due to the limitations of PCR to detect unreported or variances of ARGs, shotgun metagenomics that produce large amount of genomic information without a confined scope has been used to screen for ARGs.

The rapid development of viral metagenomics over the last decade has led to several viral metagenome (virome) analyses of various environments, including oceans, freshwater, and clinical samples [10–14], revealing a great number of novel viral sequences, including ARGs [15]. Phage-associated ARGs were found in clinical viromes [16, 17] where both bacteria and phages are heavily exposed to antibiotics, and in marine and river viromes [18–20], indicating that ARGs are commonly carried by phages in natural environments. Although a high proportion of virome reads was found to be encoding ARGs, those genes remain uncertain whether they were collected from phage genomes or bacterial genome remnants [21]. Contrast to ARG detection frequency in raw virome reads, only a few ARGs have been discovered in the viral contigs assembled from virome reads [22]. Also, in a recent study, none of the four ARGs selected from viral contigs expressed antibiotic resistance when cloned into *E. coli* [21], leading to the conclusion that numbers of phage-carried ARGs might be overestimated. Nevertheless, as ARGs continue to be detected in phage isolates and viromes from diverse environments [18, 23–26], whether phages are important reservoirs of ARGs or not is still debated.

Because only a small proportion of phages are speculated to encode ARGs, phage-associated ARGs are often neglected in ARG researches, leaving them as unsupervised resistome to threat public health. Although occurrences are rare, it is evident that phages mediate ARG transduction between bacterial cells [6] and contribute to ARG dissemination [27]. Therefore, in our present study, viral metagenomes were thoroughly studied to screen for distribution of ARGs within phage populations, search for bona fide ARGs carried by phage genomes, and verify phenotypes and functionality of the phage-encoded ARGs. Using viromes constructed from urban rivers, we performed a functional assay of ARGs recovered from viral contigs and reported the first convincing evidence of two new types of β-lactamases derived from uncultured phages. Discovery of these previously unreported ARGs in the freshwater virome suggests environmental phages could be an important reservoir of unexplored ARGs.

## Results

### Virome characteristics and antibiotic resistance genes in virome reads

**Fig. 1:**
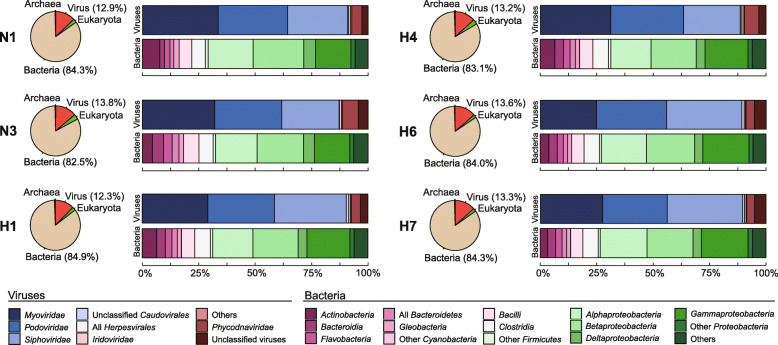
Taxonomic distribution of viral metagenome reads collected from six study sites on the Han River in South Korea. Viral taxonomy distribution is described at the family level, and bacterial taxonomy distribution is described at the class level

In May 2016, six surface water samples were collected from the Han River, South Korea (Fig. S1). Viral particles obtained from 10 L samples were subjected to metagenome sequencing using the Illumina MiSeq platform, producing 3.6 to 6.6 million reads from each site (Table S1). Among virome reads, 77.3−87.9% were annotated via the MG-RAST pipeline [28]. Only 12.3−13.8% of the annotated reads were referred to as viral reads, most of which belonged to *Myoviridae, Podoviridae,* and *Siphoviridae* of *Caudovirales* (Fig. 1), all commonly found in the environment. The majority of virome reads (82.5−84.9%) were annotated as bacterial genes that predominantly belonged to the classes *Alpha-, Beta-,* and *Gammaproteobacteria.* Since only 0.00007−0.0003% of the virome reads were found to be 16S rRNA gene sequences and proportions of single-copy bacterial marker genes were also less than 0.016% (Table S2), bacterial contamination in the virome data was negligible. Functionally annotated reads based on the SEED subsystems protein database [29] were generally annotated as phage- and prophage-related functional genes (55.6−66.7%), including terminases, capsid proteins, tail proteins, and phage DNA polymerases (Fig. S2). Of the virome reads annotated as phage-related genes, 38.6−45.0% were taxonomically assigned to bacterial genes, largely derived from *Proteobacteria* (Fig. S3). Along with phage- and prophage-related functions, virome reads were also annotated as diverse auxiliary metabolic genes (AMGs) [30], such as enzymes for carbohydrate metabolism, respiration, photosynthesis, virulence, and defenses against foreign materials (Fig. S2).

ARGs in the virome reads included genes for lactamases, multidrug transport proteins, polymyxin resistance proteins, and vancomycin resistance proteins (Table 1). Polymyxin resistance proteins were the most frequently annotated ARG, including ArnA, ArnC, and ArnT, which are involved in the lipid A-Ara4N (4-amino-4-deoxy-L-arabinose) pathway that modifies bacterial lipid cell walls to prevent the binding of cationic antibiotics [31]. Multidrug transport proteins were largely composed of ABC-type multidrug efflux pump components. Both types of ARGs, cell wall modification and multidrug efflux pump, are relatively frequently found ARGs in diverse viromes [19]. Genes for lactamases and vancomycin resistance proteins were also detected in the Han River virome at lower frequencies than other ARGs detected. Collectively, the Han River viral communities were shown to harbor ARGs at proportions of approximately 0.1%. However, the taxonomy of ARG-carrier organisms was inconclusive due to their short sequence lengths (300 bp). Short fragments of virome reads were also insufficient to anticipate whether phage-associated ARGs were incorporated into phage capsids through generalized transduction or into phage genomes through specialized transduction. Taxonomic assignment of ARG-harboring sequences was improved by assembling raw virome reads into contigs prior to further searches.

**Table 1:** Number of virome reads that were predicted to confer antibiotic resistance

Target antibiotics	H1	H3	N1	N4	N6	N7
Lactamase	11	34	61	42	77	43
Multidrug transport	45	122	49	106	61	39
Polymyxin	124	59	83	117	117	140
Vancomycin	2	4	1	0	0	2
Total	182	219	194	265	255	224
% in virome reads^a^	0.09%	0.12%	0.10%	0.09%	0.07%	0.08%

^a^[Total number of reads predicted to confer antibiotic resistance/total number of virome reads assigned a known function × 100]

### Antibiotic resistance genes in assembled viral contigs

From the six virome datasets of the Han River, raw virome reads were assembled into contigs (Materials and Methods; Table S1). Contigs carrying at least one phage-related gene were selected so that they can confidently be considered as phages genomes, resulting in 5295 viral contigs. Predicted open reading frames (ORFs) of the viral contigs were used to search for ARGs in the ARG-specific databases CARD (Comprehensive Antibiotic Resistance Database) [32], Resfams [33], and MEGARes [34], and a total of 25 ARGs were found therein (Table 2). ARG-harboring virome contigs were considered to be of viral origin (Figs. S4-S7); however, the contig sequences were not matched to any known phage genomes.

**Table 2:** List of antibiotic resistance genes retrieved from the Han River virome contigs

Contig (ORF)^a^	ARG classification	ARG	% ident.	*e* value	Query cov. (%)
H6-C148-ORF17	Antibiotic inactivation	VatA	27.39	1.93 × 10^−6^	66
H7-C136-ORF18	Antibiotic inactivation	VatA	27.39	1.93 × 10^−6^	66
N1-C442-ORF2	Antibiotic inactivation	VatB	40.91	2.42 × 10^−43^	95
H6-C980-ORF4	Antibiotic inactivation	AAC(6’)-Is	29.41	7.38 × 10^−7^	72
H4-C737-ORF5	Antibiotic inactivation	AAC(6’)-Is	29.41	7.38 × 10^−7^	72
N3-C281-ORF7	Antibiotic inactivation	AAC(6’)-Iu	30.39	8.87 × 10^−7^	72
N3-C510-ORF10	Antibiotic inactivation	AAC(6’)-Iu	31.37	8.66 × 10^−8^	72
N1-C181-ORF5	Antibiotic inactivation	AAC(6’)-Iu	30.39	8.87 × 10^−7^	72
H6-C750-ORF4	Antibiotic inactivation	AAC(6’)-Iu	31.37	9.12 × 10^−8^	72
H1-C452-ORF7	Antibiotic inactivation	AAC(6’)-Iu	30.39	8.87 × 10^−7^	72
N3-C559-ORF4	Antibiotic inactivation	AAC(6’)-Iy	20.42	5.52 × 10^−5^	84
H4-C798-ORF18	Antibiotic inactivation	AAC(6’)-Iy	20.42	5.52 × 10^−5^	84
H7-C307-ORF22	Antibiotic inactivation	AAC(6’)-Iy	20.42	5.52 × 10^−5^	84
H1-C107-ORF42	Antibiotic inactivation	AAC(6’)-Iy	20.42	5.52 × 10^−5^	84
H4-C441-ORF28^b^	Antibiotic inactivation	β-lactamase	37.18	8.00 × 10^−38^	88
H4-C244-ORF21^b^	Antibiotic inactivation	β-lactamase	37.45	1.00 × 10^−57^	96
H4-C367-ORF18^b^	Antibiotic inactivation	β-lactamase	37.45	1.00 × 10^−57^	96
H1-C74-ORF21^b^	Antibiotic inactivation	β-lactamase	37.45	1.00 × 10^−57^	96
H1-C267-ORF1	Antibiotic target protection	QnrA3	28.78	8.13 × 10^−5^	65
H6-C122-ORF31	Antibiotic target alteration	DfrB2	67.31	6.88 × 10^−21^	83
H4-C9-ORF60	Antibiotic target alteration	DfrB2	67.31	6.88 × 10^−21^	83
H7-C8-ORF44	Antibiotic target alteration	DfrB2	67.31	6.88 × 10^−21^	83
H1-C119-ORF31	Antibiotic target alteration	DfrB2	67.31	6.88 × 10^21^	83
H6-C515-ORF10	Antibiotic target alteration	VanY	27.10	1.02 × 10^−5^	76
H4-C770-ORF17	Antibiotic target alteration	VanY	27.10	1.02 × 10^−5^	76

^a^Contig number (C) and ORF number (ORF) are listed after study site number

^b^The % identity, *e* value, and query coverage of β-lactamases have been retrieved from BLASTp against NCBI nr database

Although all 25 phage-associated ARGs were selected by threshold (see Materials and Methods), it was difficult to determine the ARG functionality based solely on these sequences because most genes lacked conserved active sites or signature secondary structures and had low sequence similarities to known ARGs. The ORFs H6-C148-ORF17 and H7-C136-ORF18 encoded the *vatA* gene, and the ORF N1-C442-ORF2 encoded the *vatB* gene, the virginiamycin *O*-acetyltranferase enzymes (Table 2). The vat proteins share seven well conserved antibiotic interaction sites [35]; however, the *vatA* gene encoded by the virome contigs did not harbor any of the conserved sequences. The *vatB* gene encoded by the ORF N1-C442-ORF2 appeared to carry only four out of seven conserved residues (Fig. S8) [35], and the functionalities of VatA and VatB in the virome contigs were not able to be verified. Acetyl-CoA-dependent *N*-acetyltransferases (AAC(6’)) were found in 11 viral contigs (Table 2). The AAC(6’) in the GCN5-related *N*-acetyltransferase superfamily [36] possesses a signature secondary structure essential for acetylation of aminoglycoside antibiotics [37]. The AAC(6’) encoding genes found in 11 virome contigs had incomplete secondary structures (Fig. S9), and these enzymes were predicted to be functionally impaired. The virome contig, H1-C267, was found to encode the *qnrA3* gene that confers resistance to quinolone antibiotics. Sequences of QnrA family proteins have only a few amino acid differences among them [38], while the *qnrA3* sequence found in the viral contig had only 28.8% sequence similarity to *qnrA3* (AAZ04782.1). Additionally, it was also difficult to confirm whether this gene confers antibiotic resistance without phenotypic characterization.

Some ARGs confer antibiotic resistance through antibiotic target protein alteration, functions of which can be utilized for different purposes in bacteriophages, such as machinery for replication or infection. The dihydrofolate reductase (DHFR), *dfrB2*, was detected in four viral contigs (Table 2). The DHFR enzymes, targeted by trimethoprim, confer antibiotic resistance by providing extra DHFR gene cassette, including the *dfr*B2 genes [39, 40]. Two virome contigs were found to harbor *vanY* genes (Table 2) encoding the d-alanyl-d-alanine carboxypeptidase that cleaves d-Ala from peptidoglycan precursors to prevent the binding of vancomycin [41] and also known to function as a bacteriophage endolysin [42]. Thereby, antibiotic resistance function of *dfrB2* and *vanY* genes in bacteriophages could not be definitively determined. On the contrary, β-lactamases encoded by four virome contigs (Table 2) had clear indication of antibiotic resistance function due to well-conserved active sites (presented below). We thereby focused our detailed functional analysis on these four β-lactamase genes.

### Novel and functional β-lactamases encoded by viral contigs

**Fig. 2:**
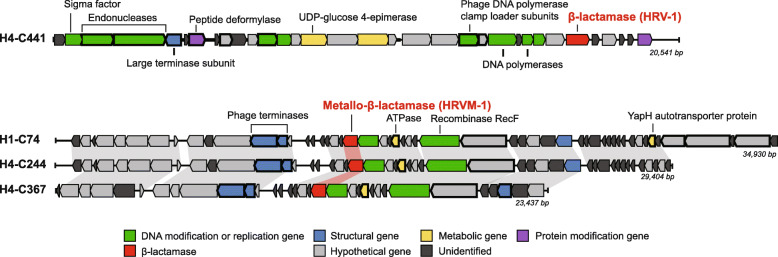
Sequence maps of contigs H4-C441, H1-C74, H4-C244, and H4-C367-bearing β-lactamase genes HRV-1 or HRVM-1. Red, HRV-1 or HRVM-1; green, ORFs coding DNA modification or replication; blue, bacteriophage structural genes; purple, ORFs related to protein modification; yellow, metabolic genes; light grey, hypothetical genes or predicted organisms; dark grey, unidentified ORFs. The genes outlined in bold indicate viral origin

**Fig. 3:**
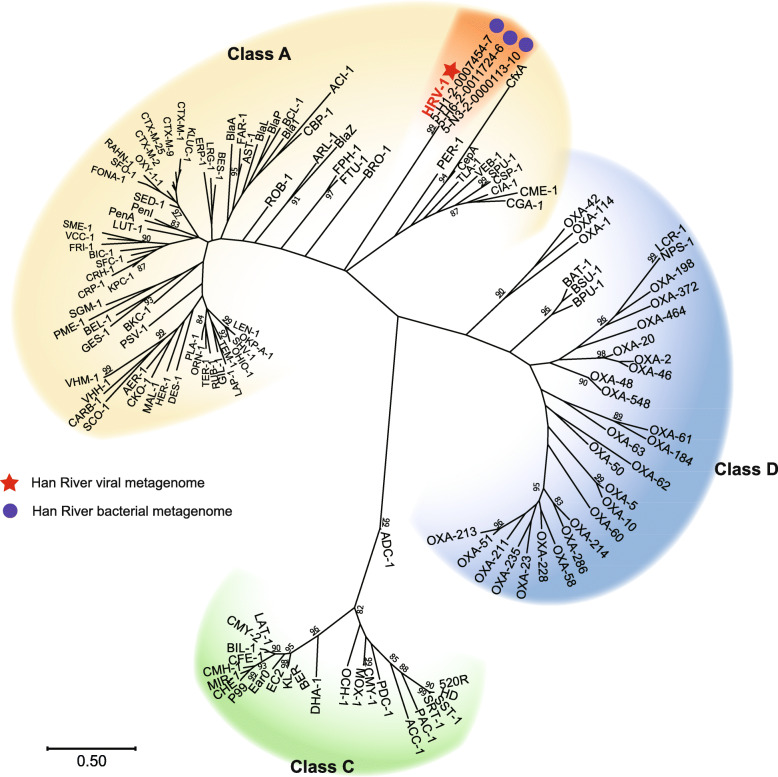
Maximum-likelihood phylogenetic tree of HRV-1 with representative enzymes of classes A, C, and D β-lactamases. Numbers at the nodes represent bootstrap values based on 100 re-samplings, and values higher than 80% are shown. Metagenome sequences used for the tree construction, marked with blue circles, were selected from the Han River bacterial metagenome. Accession numbers for the representative sequence types of β-lactamases used for phylogenetic analyses are listed in Table S3

**Fig. 4:**
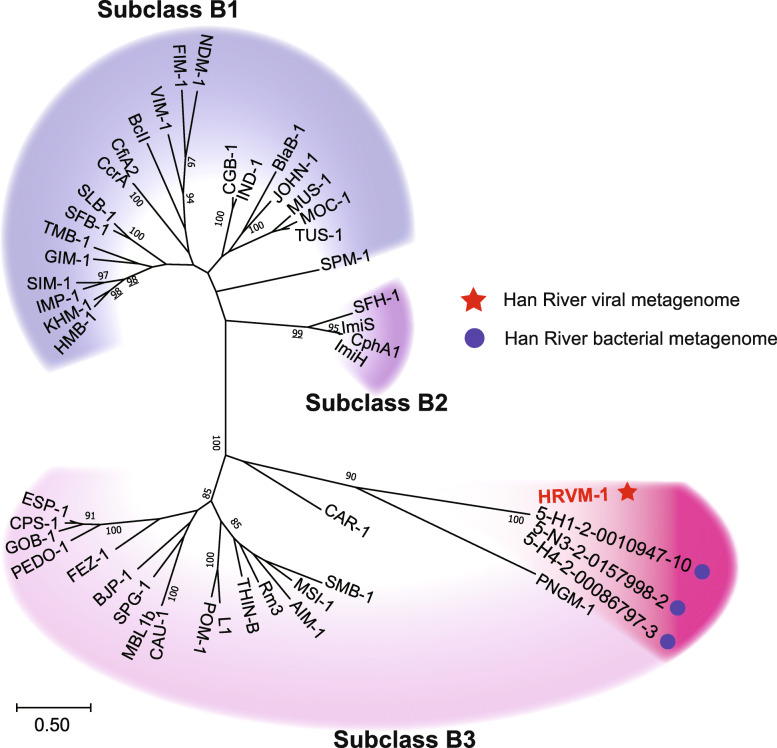
Maximum-likelihood phylogenetic tree of HRVM-1 with representative enzymes of subclasses B1, B2, and B3. Numbers at the nodes represent bootstrap values based on 100 re-samplings, and values higher than 80% are shown. Metagenome sequences used for the tree construction, marked with blue circles, were selected from the Han River bacterial metagenome. Accession numbers for the representative sequence types of β-lactamases used for the phylogenetic analyses are listed in Table S3

β-lactamases, one of the widely found ARGs in diverse environments, were found in four viral contigs (Fig. 2). Among these, H4-C441-ORF28 was related to class A β-lactamases and three ORFs (H1-C74-ORF21, H4-C244-ORF21, and H4-C367-ORF18), sharing identical nucleotide sequences and showed homology to metallo-β-lactamases (MBLs, Table 2). The ORF H4-C441-ORF28 carried the conserved active sites S_70_-X-X-K_73_ and S_135_-D_136_-N_137_ [43], the motifs specific to class A β-lactamases (Fig. S10). Phylogenetic analyses also showed that H4-C441-ORF28 was affiliated with class A β-lactamases but formed a distinctive clade apart from representative class A β-lactamases (Fig. 3, Table S3). Accordingly, this unique class A β-lactamase gene was named *bla*_HRV-1_, and its product was named HRV-1 (Han River Virome β-lactamase-1). Three ORFs, H1-C74-ORF21, H4-C244-ORF21, and H4-C367-ORF18, were predicted to be related to MBLs with the conserved active site, H_147_-X-H_149_-X-D_151_-H_152_ [44] (Fig. S11). A maximum-likelihood phylogenetic tree grouped the MBL-related ORFs into subclass B3 MBLs, also forming an independent monophyletic cluster (Fig. 4, Table S3); this novel gene and gene product were named as *bla*_HRVM-1_ and HRVM-1 (Han River Virome Metallo-β-lactamase-1), respectively. The presence of conserved active sites of β-lactamases suggested the possibility of their functionality; therefore, phenotype analyses were performed on HRV-1 and HRVM-1.

**Fig. 5:**
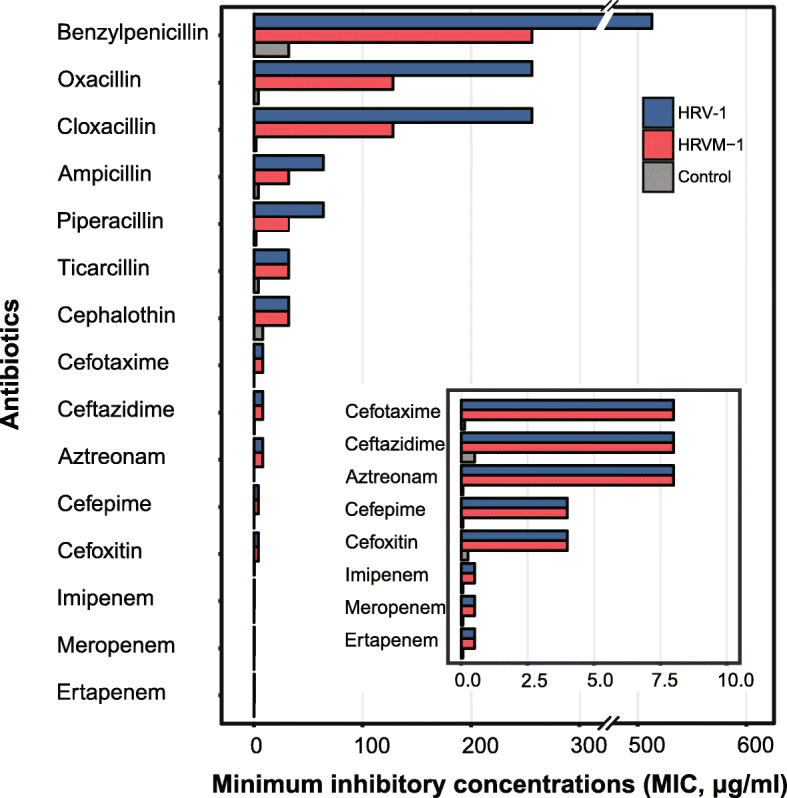
Minimum inhibitory concentrations (MICs) of β-lactams for *Escherichia coli* BL21 (DE3) transformants producing HRV-1 [*E. coli* BL21 (DE3)-pET-30a(+)-HRV-1] or HRVM-1 (*E. coli* BL21 (DE3)-pET-28a(+)-HRVM-1) or harboring the expression vectors pET-28a(+) or pET-30a(+) [*E. coli* BL21 (DE3)-pET-28a(+)/*E. coli* BL21 (DE3)-pET-30a(+)]. The inset graph is a magnification of MIC values of 8 or less

For the phenotypic expression analysis, pET-30a(+)/*bla*_HRV-1_-His_6_ and pET-28a(+)/*bla*_HRVM-1_-His_6_ plasmids were transformed into *E. coli* BL21 (DE3), and their susceptibilities to β-lactam antibiotics were assessed. The two strains showed reduced susceptibility, ranging from 2- to 16-fold reductions, to extended-spectrum cephalosporins and carbapenems, as well as to penicillin and narrow-spectrum cephalosporins, displaying typical characteristics of extended-spectrum β-lactamases (ESBL) or carbapenemase (Fig. 5 and Table S4). A kinetic analysis of purified HRV-1 and HRVM-1 revealed that these two enzymes had broad substrate profiles including carbapenems (Table S5). The catalytic efficiencies (*k*_*cat*_*/k*_*m*_) of HRV-1 for cefotaxime (4.1 × 10^3^ M^−1^ S^−1^) and ceftazidime (3.3 × 10^3^ M^−1^ S^−1^) were 2- and 26-fold higher than those of GES-14 (1.8 × and 0.125 × 10^3^ M^−1^ S^−1^, respectively) belonging to class A ESBL [45]. In addition, the *k*_*cat*_*/k*_*m*_ of HRVM-1 for imipenem (5.0 × 10^2^ M^−1^ s^−1^) was similar to those of PNGM-1 (5.5 × 10^2^ M^−1^ s^−1^) [46] from a deep-sea sediment metagenome and CAR-1 (9.6 × 10^2^ M^−1^ s^−1^) [47] from *Erwinia carotovora* that belong to subclass B3 MBL. The kinetic data for HRV-1 and HRVM-1 were consistent with the determined MICs (Fig. 5 and Table S4), indicating that HRV-1 and HRVM-1 have ESBL and carbapenemase properties.

**Fig. 6:**
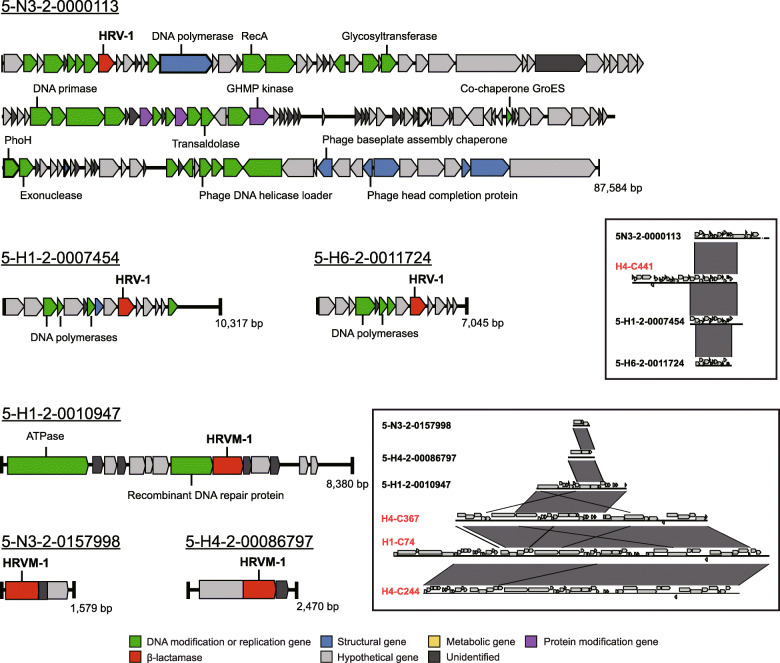
Genomic maps of the Han River bacterial metagenome contigs that harbor homologous ORFs to HRV-1 or HRVM-1. Red, HRV-1 or HRVM-1; blue, ORFs with virus-related functions; green, ORFs with general metabolic function; grey, hypothetical proteins. The boxed areas show a simplified depiction of the sequence synteny between viral and bacterial metagenome contigs that carry HRV-1 or HRVM-1

In addition, bacterial metagenome datasets were generated in parallel at identical sampling stations. Homologous sequences to HRV-1 and HRVM-1 were searched within the metagenome-assembled contigs to observe their occurrence in bacterial fractions. Three metagenomic ORFs, each with 100% amino acid sequence identity, were found for both HRV-1 and HRVM-1, (Figs. 3 and 4; Tables S6 and S7). Although these six contigs were discovered within bacterial metagenome sequences, they were predicted to be viral genomes (scores 0.954–0.999 from DeepVirFinder [48]). Furthermore, the metagenome contigs with these ORFs showed high synteny to the viral contigs containing *bla*_HRV-1_ and *bla*_HRVM-1_ (Fig. 6), suggesting the presence of infectious phages or prophages carrying *bla*_HRV-1_ and *bla*_HRVM-1_ in the Han River bacterial communities [49]. Public viral and bacterial metagenomes were additionally searched to assess the global distribution of HRV-1 and HRVM-1. Sequences similar to HRV-1 (amino acid sequence similarity up to 69%) were frequently found in metagenomes prepared from the Colombia River estuary in the USA, while HRVM-1-like sequences (amino acid sequence similarity up to 71%) were mainly found in freshwater viromes from Singapore (Tables S6 and S7 and Fig. S12), suggesting that HRV-1 and HRVM-1 are globally distributed. In particular, *bla*_HRVM-1_ showed a sequence similarity of ~ 40% to metallo-hydrolases found in *Clostridium botulinum* and *Clostridioides difficile* (Table S7), imposing that HRVM-1 and its homologous sequences are possibly carried by phages that infect these pathogens.

## Discussion

Diverse ARGs have been detected in phage DNA fractions or virome reads recovered from various environments including freshwater [50], wastewater treatment plants [8], ocean [18], and animal feces [26]. Despite continued reports of phage-associated ARGs, only few of those genes have been evaluated for antibiotic resistance capacity, and of these, most were shown to be non-functional [21]. Therefore, whether ARGs recovered from different viromes are truly functional or not has been continuously questioned. In this study, we conducted an in-depth study on ARGs excavated from urban river viromes and discovered phage-associated ARGs, including two novel and functional β-lactamases, providing the solid evidence that viromes encompass genuine functional ARGs. This new finding alerts us of the existence of numerous unexplored functional ARGs in environmental viral populations that have thus far been overlooked due to an absence of functionality validation.

Although the Han River viromes were specifically targeted for phage particles, most of the reads were predicted to be bacterial (Fig. 1). The annotation of virome reads as bacterial genes is a common phenomenon due to dearth of viral gene database as most of phages in the environment are not yet cultured. Hence often, virome reads encoding bona fide viral proteins are improperly represented as bacterial genes. As infecting uncultured phages or prophages get sequenced along with their host bacterial genomes, their genes can be reported as phage functional gene with bacterial taxonomy, also leading to misinterpretation of virome reads. Although most of the Han River virome reads were taxonomically predicted to be bacterial sequences, approximately 62% of them were functionally annotated to encode phage-related proteins (Fig. S2). Therefore, we can speculate that the Han River virome reads that were assigned to bacterial taxonomy are in fact, viral sequences. In addition, bacterial contamination evaluation revealed that the virome data harbored very low percentage of bacterial marker genes such as SSU and LSU rRNA genes; hence, the viromes were unlikely to be contaminated with bacterial cells (Table S2). Therefore, virome reads predicted to have bacterial function, such as ARGs, were considered as viral origin.

ARGs developed in bacterial cells as a defense mechanism against antibiotic-producing microorganisms or antibiotics and phages have coincidently acquired these genes through sporadic recombination with host genomes during infection [23]. Therefore, only a small number of ARGs are expected to be found in viral sequences. Within the six viromes generated in our present study, 0.07–0.12% of the reads were predicted to be ARG sequences (Table 1). The proportions of ARGs in the viromes were comparable with ARG levels detected from other study sites. River viromes collected from the Minnesota, USA, appeared to contain approximately 0.13% of the virome reads as ARGs when analyzed using the MG-RAST annotation pipeline [50]. When multiple freshwater virome reads were evaluated for ARGs using the ARG-specific databases PATRIC and Resfams, only about 0.001% of the reads were predicted to encode ARGs [24]. In other environmental viromes, such as those prepared from soil or ocean samples, a more variable but still low proportion of virome reads were annotated as ARGs, ranging from 0.001 to 0.440% [18, 24, 50]. As previously claimed [21], phages, as well as viromes, are likely to carry and encode ARGs only at rare events.

The Han river flows from more pristine area (N1 and N3) to an urbanized area (H1, H4, H6, and H7) and as anthropogenic influences on the river increased, ARG levels in viral populations were expected to increase concomitantly. However, we observed no significant differences in ARG levels among the six samples from varying areas (Table 1). Similar trend was observed in the Lambro River, Italy [19]. Virome samples collected from a pristine area on the Lambro River appeared to have the highest level of ARGs (1.92%), while that collected from an urbanized site showed the lowest level of ARGs (0.48%). ARGs have been also continuously detected at low frequency (0.08–0.22%) in marine viromes generated from open oceans, where anthropogenic influences are at its minimum [18]. These findings suggest that phage-associated ARGs are a rare natural phenomenon rather than human-activity attributed event.

When ARGs are horizontally transferred through phages, they can be either encapsulated in phage particles as gene fragments or incorporated into phage genomes. Although ARGs within phage particles can be fully detected through screening of virome reads, whether these ARGs are encoded by phage genomes or not is difficult to determine. Therefore, the screening of ARGs in long viral contigs, which resemble putative phage genomes, was necessary to search for ARGs specifically encoded by phages. Among 5295 viral contigs constructed in this study, only 25 contigs were found to encode ARGs (Table 2). As seen in ARG screening analyses of the raw virome reads, only a small number of viral genomes were shown to carry ARGs. Enault and colleagues [21] have reported that phage-associated ARGs are highly rare, and even if detected, the ARGs are unlikely to confer antibiotic resistance, concluding that most of the ARGs observed in virome reads might be caused by bacterial gene contamination. For the Han River virome data, the bacterial contamination was negligible in virome reads-level (Table S2). In addition, virome contigs used for the ARG analyses in this study were selected based on the presence of virus-specific genes and characteristics. We can thereby confidently claim that the small number of ARGs detected in this study is of viral origin without bacterial contamination.

We found four viral ORFs encoding β-lactamases that confer antibiotic resistance against β-lactam antibiotics, including carbapenems (Figs. 2 and 5). These four β-lactamase genes were identified by the Resfams database but not by the CARD or MEGARes databases. The Resfams database includes most of the representative ARGs and especially focuses on β-lactamase variants [33]. Therefore, the combined use of the Resfams, CARD, and MEGARes databases to screen for ARGs allowed us to detect diversified and novel β-lactamase genes from viral contigs. Compared to other previous researches to screen for ARGs within virome data [15, 21, 22, 51, 52], we have employed lenient cutoffs (*e* value of ≤ 1 × e^−5^ and query coverage of ≥ 60% or gathering cutoff) in refining ARG search results. When conservative thresholds that have been used in the previous researches were applied (amino acid sequence identity ≥ 80% and query coverage ≥ 85%), no ARG was detected within the Han River virome contigs (Table S8). With relaxed thresholds used in this study, we discovered multiple virome ORFs showing homology to ARGs with conserved active sites including two novel β-lactamases. To the best of our knowledge, the two novel β-lactamase genes discovered in the present study, *bla*_HRV-1_ and *bla*_HRVM-1_, are the first ARGs discovered from viral metagenome contigs that confer true antibiotic resistance. β-Lactamases are bacterial enzymes that hydrolyze the β-lactam ring of β-lactam antibiotics and have been implicated in the emergence of antibiotic-resistant pathogenic bacteria [53, 54]. Novel β-lactamase genes are continuously being found in clinical and environmental bacterial populations [55–57], threatening patients with potentially untreatable pathogenic infections. The presence of these two novel β-lactamase genes in freshwater viromes suggests another threat to public health. Phages are small, abundant, ubiquitous, diverse, and polyvalent, and are difficult to track, making it challenging to predict the dissemination routes of these phage-borne ARGs. Phage-associated ARGs pose a significant threat also because phage capsids are more persistent against wastewater treatments [58], allowing safe transportation into bacterial hosts. The presence of two novel β-lactamases in the freshwater virome indicates that phages are ARG carriers along with bacterial cells, and that phage populations could be significant ARG reservoirs. Vast amounts of virome data that were bypassed, or evaluated for encompassing ARG sequences only, might contain a broad range of ARGs and should be revisited for further evaluations.

Metagenome-assembled genomes (MAGs) of bacteria and viruses have contributed to expanding our knowledge of microbial populations that have yet to be cultured [59]. Genes and genomes predicted from metagenome data often have highly variable sequences with low sequence similarity to previously identified reference sequences. A variety of novel ARGs with distinctive sequences have been discovered from bacterial metagenomes [60]. The novel β-lactamase genes, *bla*_RSA_, *bla*_RSC_, and *bla*_RSD_, were all recovered from soil bacterial metagenomes [61]. Especially, the genes *bla*_RSD1_ and *bla*_RSD3_ had significantly low sequence similarities (≤ 45%) to previously reported β-lactamases, raising questions about their functionality, but were ultimately shown to confer antibiotic resistance with high MICs. Novel quinolone resistance genes (*qnr*) recovered from Tara ocean metagenomes [62] also had low sequence similarity (≤ 37%) to their best matches, but most of them (24 out of 27) showed increased MICs of ciprofloxacin. In the present study, we also discovered novel ARGs from viral metagenomes with high sequence variability. In addition to the four ORFs encoding *bla*_HRV-1_ and *bla*_HRVM-1_, we also found 21 ORFs encoding other ARGs, such as *vatA*, *aac*, *qnr*, *dfrB*, and v*anY* (Table 2). Since requisite active sites or core secondary structures of these enzymes were not predictable by sequence alignment analysis only, the ORFs were excluded from functional analyses in this study. However, as shown by the sequences of *bla*_HRV-1_ and *bla*_HRVM-1_ which showed antibiotic resistance even with low sequence similarity to the best matches (37%), the ARGs predicted by 21 ORFs may also confer antibiotic resistance when their functions are experimentally tested. Therefore, all ARGs predicted from this study require further functional studies to determine their resistance capacities.

Phages are generally known to have small and highly diversified genomes caused by fast recombination and mutation events [63, 64]. After frequent exposure to genetic alterations such as HGTs, genes essential and beneficial to phage particles are likely to be maintained in phage genomes. The β-lactamases HRV-1 and HRVM-1 that we discovered are not considered as temporarily carried genes in phage capsids after general transduction but stably incorporated transduced genes through specialized transduction. Observation of HRV-1 and HRVM-1 sequences in different virome data indicated that these genes could be carried across and maintained within phage populations. The findings of β-lactamases in the phage genomes suggest that these genes may be utilized as AMGs during phage infection. AMGs are bacterial metabolic genes carried by phage genomes and expressed during infection to the benefit of both phages and hosts [30]. One of the most widely known phage AMGs is the *psaA* gene of photosystems I and II, which is expressed during infection to complement the hosts’ photosystem and enhance host cell metabolism to increase phage genome replication [65]. Likewise, the ARGs, HRV-1 and HRVM-1, could be carried by short phage genomes to be utilized as AMGs to contribute to host cell survival under antibiotic stress.

All 25 virome contigs harboring ARGs were predicted to be of viral origin, but neither viral nor host taxonomy of these contigs could be determined due to the uniqueness of the sequences. Most of the viral contigs that we discovered from the virome data are thus considered to originate from the genomes of uncultured and undiscovered phages. The HRVM-1 protein sequence showed homology with other metallo-hydrolases encoded by the human pathogens *Clostridium botulinum* and *Clostridioides difficile*. Although taxonomic and host information for the HRVM-1-harboring viral contig is not definable, the potentials for HRVM-1 to be transferred between pathogens and even cause antibiotic-resistant pathogenic infection are not negligible. This discovery of two new functional and novel β-lactamases, HRV-1 and HRVM-1, from the freshwater virome suggests the presence of an unknown environmental bacteriophage community that could pose an additional threat to antibiotic treatments.

## Conclusion

This study was performed in natural environments where anthropogenic influences were minimal or moderate and nonetheless discovered novel β-lactamases that confer resistance against diverse β-lactam antibiotics. More ARGs with higher sequence diversity are expected to be found in environmental regimes subject to greater antibiotic stress. The discovery of HRV-1 and HRVM-1 from virome data shows that despite being rarely encountered, phage-encoded ARGs can confer antibiotic resistance and possess unique sequences that could be introduced into host bacterial cells. Our results suggest that viromes are important reservoirs of ARGs and hitherto unrecognized β-lactamases and show how phages contribute to the dissemination of antibiotic resistance and present a potential threat to human health. Therefore, we recommend that the vast repository of virome sequences accumulated from diverse environments be studied more extensively for the excavation and identification of functional and novel ARGs that may have been overlooked in the past.

## Methods

### Sequencing of the Han River viral metagenomes

In May 2016, 10 L of surface water was collected from each of six stations on the Han River, South Korea (Fig. S1). The samples were filtered through a GF/A glass microfiber filter (Whatman, Maidstone, UK) followed by a 0.2-μm Supor® PES Membrane filter (Pall Corporation, New York, New York, US) to remove prokaryotic cells. The viral particles in the filtrates were flocculated by adding 0.01 g of FeCl_3_·6H_2_O per 10 L of filtered sample, incubated at room temperature for 12 h, and collected on 0.8-μm Isopore polycarbonate filters (Merck Millipore, Burlington, MA, USA) [66]. The filters were dissolved in 0.1 M EDTA-0.2 M MgCl_2_-0.2 M ascorbate buffer and treated with DNase I and RNase A at final concentrations of 10 U/mL and 1 U/mL (Sigma-Aldrich, St. Louis, MO, USA), respectively, to remove any external nucleic acids [67]. The concentrated viral particles were purified through CsCl step-gradient ultracentrifugation [68] and subjected to viral DNA extraction using the DNeasy Blood and Tissue Kit (Qiagen, Hilden, Germany). The DNA samples were used for TruSeq library construction and sequenced using the Illumina MiSeq platform, with 2 × 300-bp paired-end reads (ChunLab Inc., Seoul, Korea). The raw sequencing reads were screened using Trimmomatic [69] based on quality score and length (LEADING:10 TRAILING:10 SLIDINGWINDOW:4:16 MINLEN:100). The virome reads were submitted to the MG-RAST metagenome analysis server (www.metagenomics.anl.gov) for analysis [28]. Taxonomic and functional annotations of virome reads were performed with the MG-RAST analysis pipeline using a non-redundant M5nr database [70], with a threshold of *e* value ≤ 1 × e−^5^ and percent identity ≥ 60% over a minimum sequence length of 15 amino acids. The presence of bacterial contamination in the virome samples was evaluated by calculating the proportion of bacterial 16S rRNA genes and single-copy bacterial genes using the METAXA [71] and ViromeQC programs (-w environmental) [72]. All virome reads were assembled into contigs using SPAdes version 3.8.2 [73] with the *metaspades.py* option (-k 27,47,67,87,107,127). Among the assembled contigs, only those longer than 10 kbp and those containing viral protein-coding sequences (CDS) were used for further analyses (Table S1).

### Antibiotic resistance gene search and sequence analysis

The finalized contigs were predicted to be of viral (herein refers to phage or archaeal virus) or bacterial origin based on the presence of viral hallmark genes or high proportion of hypothetical genes, which are the typical characteristics of environmental viral contigs, using the VirSorter program with the “Virome” database and “virome decontamination” option [74]. All virome contigs classified as VirSorter categories 1 (“most confident”), 2 (“likely”), and 3 (“possible” phage genomes) were accepted for further analyses. The CDS of virome ORFs were predicted using the VirSorter program, and those were used as queries to search for ARGs using BLASTp [75] against the CARD or MEGARes with thresholds of *e* value ≤ 1 × e^−5^ and query coverage ≥ 60% and hmmscan [76] against the Resfams ARG database [33], with a cutoff of *e* value ≤ 1 × e^−5^ and a gathering threshold score ≥ 40. The results were manually curated to remove ARGs that confer antibiotic resistance by single nucleotide mutations. Curated searches revealed 25 CDSs encoding an ARG. Multiple sequence alignments of the CDSs were performed using Clustal Omega [77] with respective representative ARGs. Maximum-likelihood phylogenic trees with JTT-model were generated with MEGA 7 [78], and the tree topologies were evaluated by bootstrap analyses based on 100 re-samplings.

### Cloning and antimicrobial susceptibility tests of HRV-1 and HRVM-1 genes

The MICs of HRV-1 and HRVM-1 were assessed with PCR to derive their sequences using the primer pairs listed in Table S9. The PCR products were double-digested with *Nde*I and *Xho*I for the HRV-1 and *Nco*I and *Xho*I for the HRVM-1, and the digested DNAs were cloned into corresponding restriction enzyme-digested pET-28a(+) or pET-30a(+) vectors (Novagen, Madison, WI, USA). Recombinant plasmids [pET-30a(+)/*bla*_HRV-1_-His_6_ and pET-28a(+)/*bla*_HRVM-1_-His_6_] were transformed into *E*. *coli* BL21 (DE3) (Invitrogen, Carlsbad, CA, USA).

When cultures of the cloned *E. coli* BL21 (DE3) cells reached approximately 0.6 at OD_600_, 1 mM isopropyl β-D-1-thiogalactopyranoside was added to induce expression of the *bla*_HRV-1_ or *bla*_HRVM-1_ gene followed by culture at 16 °C for 16 h. Susceptibility to benzylpenicillin, ampicillin, piperacillin, ticarcillin, oxacillin, cloxacillin, cephalothin, cefoxitin, cefotaxime, ceftazidime, cefepime, aztreonam, meropenem, imipenem (Sigma-Aldrich), and ertapenem (Merck & Co., Inc, Kenilworth, NJ, USA) against cloned *E*. *coli* BL21 (DE3) was assessed using the agar dilution technique in Mueller-Hinton agar (Difco Laboratories Inc., Detroit, MI, USA) including 1 mM IPTG with an inoculum of 10^5^ CFU per spot [79].

### Expression and purification of recombinant HRV-1 and HRVM-1

The *bla*_HRV-1_ and *bla*_HRVM-1_ genes were expressed by the addition of IPTG, and their proteins were purified by running the cell lysate through a His-Bind column (Novagen), and the His_6_ tag was then removed by the addition of enterokinase according to the manufacturer's instructions (Novagen). The mixture was re-purified using Fast Desalting and Mono S columns (Amersham Biosciences, Little Chalfont, UK). Protein purity was examined by SDS-PAGE. Soluble forms of homogeneous HRV-1 and HRVM-1 were obtained with yields of 4.4 and 3.4 mg per liter of culture, and their molecular weights were estimated to be 26.2 and 27.6 kDa, respectively.

Kinetic assays for purified HRV-1 and HRVM-1 were conducted at 30 °C with a Shimadzu UV-1650PC spectrophotometer (Shimadzu Corp., Kyoto, Japan). β-Lactam hydrolysis was assessed by monitoring variations in absorbance using the characteristic molecular extinction coefficient of each substrate (Table S10). Assays of HRV-1 were conducted in 10 mM MES [2-(N-morpholino) ethanesulfonic acid] buffers (pH 6.8) containing enzymes (10–1000 nM) and 20 μg/mL bovine serum albumin. Those of HRVM-1 were conducted in 50 mM MES (pH 7.0) containing enzymes (13–1200 nM), 100 μM ZnCl_2_, and 100 μg/mL bovine serum albumin. Steady-state kinetic constants were determined by fitting the initial rates (in triplicate) directly to the Henri-Michaelis-Menten equation using a nonlinear regression with the DynaFit [80] program.

### Analysis of HRV-1 and HRVM-1 genes in bacterial metagenomes

A bacterial metagenome concurrent with the viral metagenome was constructed with the same water samples used for virome generation. The water samples were pre-filtered through a 10 μm pore-sized nylon membrane (Merck Millipore) followed by the collection of microbial biomasses onto a 0.2 μm pore-sized mixed cellulose ester membrane (Advantec, Taipei, Taiwan). Metagenomic DNA was extracted from the membranes using the DNeasy PowerWater DNA Isolation Kit (Qiagen). Shotgun metagenome sequencing was performed using the Illumina HiSeq 4000 platform (ChunLab Inc.) which yielded approximately 21 Gb of 2 × 150-bp paired-end reads from each sample, followed by sequence quality control by FaQCs [81]. The metagenome assembly was performed using the IDBA-UD v1.1 [82]. CDSs in the metagenome contigs were predicted by prodigal v2.6 [83] with the -*p meta* option. A BLASTp search was performed against the database constructed using the predicted CDSs of the bacterial metagenome contigs (cutoffs of sequence identity ≥ 50%, reference coverage ≥ 80%, and *e* value ≤ 1 × e^−4^) to search for genes related to HRV-1 and HRVM-1 in the bacterial metagenomes. HRV-1 and HRVM-1-like genes were further searched in metagenome databases using the HMMER search engine (www.ebi.ac.uk/metagenomics/) with an e-value cutoff of ≤ 1 × e^−30^. The bacterial metagenome contigs found to be carrying either HRV-1 or HRVM-1 sequences were determined whether virus-related using the DeepVirFinder program [48] with default parameters.

## Supplementary information

**Additional file 1: Tables S1-S10** and **FiguresS1-S14.**

## Data Availability

The viral metagenome sequences obtained from this study have been deposited in the EBI database under the accession number ERP021385. The contig sequences containing β-lactamases are available in the EBI database under the following accession numbers: ERS2955525 for H1-C74, ERS2955526 for H4-C244, ERS2955527 for H4-C367, and ERS2955528 for H4-C441.

## References

[1] World Health Organization (WHO) (2018). Global Antimicrobial Resistance Surveillance System (GLASS) Report: Early implementation 2017-2018.

[2] Berglund B (2015). Environmental dissemination of antibiotic resistance genes and correlation to anthropogenic contamination with antibiotics. Infect Ecol Epidermiol.

[3] von Wintersdorff CJH, Penders J, van Niekerk JM, Mills ND, Majumder S, van Alphen LB (2016). Dissemination of antimicrobial resistance in microbial ecosystems through Horizontal Gene Transfer. Front Microbiol.

[4] Yu P, Mathieu J, Li M, Dai Z, Alvarez PJ (2016). Isolation of polyvalent bacteriophages by sequential multiple-host approaches. Appl Environ Microbiol.

[5] Brown-Jaque M, Calero-Cáceres W, Muniesa M (2015). Transfer of antibiotic-resistance genes via phage-related mobile elements. Plasmid..

[6] Modi SR, Lee HH, Spina CS, Collins JJ (2013). Antibiotic treatment expands the resistance reservoir and ecological network of the phage metagenome. Nature..

[7] Brown-Jaque M, Calero-Cáceres W, Espinal P, Rodríguez-Navarro J, Miró E, González-López JJ (2017). Antibiotic resistance genes in phage particles isolated from human feces and induced from clinical bacterial isolates. Int J Antimicrob Agents.

[8] Colomer-Lluch M, Jofre J, Muniesa M (2014). Quinolone resistance genes (qnrA and qnrS) in bacteriophage particles from wastewater samples and the effect of inducing agents on packaged antibiotic resistance genes. J Antimicrob Chemother.

[9] Lekunberri I, Villagrasa M, Balcázar JL, Borrego CM (2017). Contribution of bacteriophage and plasmid DNA to the mobilization of antibiotic resistance genes in a river receiving treated wastewater discharges. Sci Total Environ.

[10] Angly FE, Felts B, Breitbart M, Salamon P, Edwards RA, Carlson C (2006). The marine viromes of four oceanic regions. PLoS Biol.

[11] Brum JR, Ignacio-Espinoza JC, Roux S, Doulcier G, Acinas SG, Alberti A (2015). Patterns and ecological drivers of ocean viral communities. Science..

[12] Roux S, Enault F, Robin A, Ravet V, Personnic S, Theil S (2012). Assessing the diversity and specificity of two freshwater viral communities through metagenomics. PLoS One.

[13] Skvortsov T, de Leeuwe C, Quinn JP, McGrath JW, Allen CC, McElarney Y (2016). Metagenomic characterisation of the viral community of Lough Neagh, the largest freshwater lake in Ireland. PLoS One.

[14] Yutin N, Makarova KS, Gussow AB, Krupovic M, Segall A, Edwards RA (2017). Discovery of an expansive bacteriophage family that includes the most abundant viruses from the human gut. Nat Microbiol.

[15] Subirats J, Sànchez-Melsió A, Borrego CM, Balcázar JL, Simonet P (2016). Metagenomic analysis reveals that bacteriophages are reservoirs of antibiotic resistance genes. Int J Antimicrob Agents.

[16] Brown-Jaque M, Rodriguez Oyarzun L, Cornejo-Sánchez T, Martín-Gómez MT, Gartner S, de Gracia J (2018). Detection of bacteriophage particles containing antibiotic resistance genes in the sputum of cystic fibrosis patients. Front Microbiol.

[17] Colomer-Lluch M, Imamovic L, Jofre J, Muniesa M (2011). Bacteriophages carrying antibiotic resistance genes in fecal waste from cattle, pigs, and poultry. Antimicrob Agents Chemother.

[18] Calero-Cáceres W, Balcázar JL (2019). Antibiotic resistance genes in bacteriophages from diverse marine habitats. Sci Total Environ.

[19] Colombo S, Arioli S, Neri E, Della Scala G, Gargari G, Mora D (2017). Viromes as genetic reservoir for the microbial communities in aquatic environments: a focus on antimicrobial-resistance genes. Front Microbiol.

[20] Colomer-Lluch M, Jofre J, Muniesa M (2011). Antibiotic resistance genes in the bacteriophage DNA fraction of environmental samples. PLoS One.

[21] Enault F, Briet A, Bouteille L, Roux S, Sullivan MB, Petit M-A (2017). Phages rarely encode antibiotic resistance genes: a cautionary tale for virome analysis. ISME J.

[22] Lekunberri I, Subirats J, Borrego CM, Balcázar JL (2017). Exploring the contribution of bacteriophages to antibiotic resistance. Environ Pollut.

[23] Calero-Cáceres W, Ye M, Balcázar JL (2019). bacteriophages as environmental reservoirs of antibiotic resistance. Trends Microbiol.

[24] Debroas D, Siguret C (2019). Viruses as key reservoirs of antibiotic resistance genes in the environment. ISME J.

[25] Larrañaga O, Brown-Jaque M, Quirós P, Gómez-Gómez C, Blanch AR, Rodríguez-Rubio L (2018). Phage particles harboring antibiotic resistance genes in fresh-cut vegetables and agricultural soil. Environ Int.

[26] Wang M, Liu P, Zhou Q, Tao W, Sun Y, Zeng Z (2018). Estimating the contribution of bacteriophage to the dissemination of antibiotic resistance genes in pig feces. Environ Pollut.

[27] Balcázar JL (2018). How do bacteriophages promote antibiotic resistance in the environment?. Clin Microbiol Infect.

[28] Meyer F, Paarmann D, D'Souza M, Olson R, Glass EM, Kubal M (2008). The metagenomics RAST server - a public resource for the automatic phylogenetic and functional analysis of metagenomes. BMC Bioinformatics.

[29] Overbeek R, Olson R, Pusch GD, Olsen GJ, Davis JJ, Disz T (2014). The SEED and the Rapid Annotation of microbial genomes using Subsystems Technology (RAST). Nucleic Acids Res.

[30] Hurwitz BL, U’Ren JM (2016). Viral metabolic reprogramming in marine ecosystems. Curr Opin Microbiol.

[31] Olaitan AO, Morand S, Rolain J-M (2014). Mechanisms of polymyxin resistance: acquired and intrinsic resistance in bacteria. Front Microbiol.

[32] Jia B, Raphenya AR, Alcock B, Waglechner N, Guo P, Tsang KK (2017). CARD 2017: expansion and model-centric curation of the comprehensive antibiotic resistance database. Nucleic Acids Res.

[33] Gibson MK, Forsberg KJ, Dantas G (2015). Improved annotation of antibiotic resistance determinants reveals microbial resistomes cluster by ecology. ISME J.

[34] Lakin SM, Dean C, Noyes NR, Dettenwanger A, Ross AS, Doster E (2017). MEGARes: an antimicrobial resistance database for high throughput sequencing. Nucleic Acids Res.

[35] Stogios PJ, Kuhn ML, Evdokimova E, Courvalin P, Anderson WF, Savchenko A (2014). Potential for reduction of streptogramin A resistance revealed by structural analysis of acetyltransferase VatA. Antimicrob Agents Chemother.

[36] Favrot L, Blanchard JS, Vergnolle O (2016). Bacterial GCN5-related N-Acetyltransferases: from resistance to fegulation. Biochemistry..

[37] Vetting MW (2005). LP S d C, Yu M, Hegde SS, Magnet S, Roderick SL, Blanchard JS. Structure and functions of the GNAT superfamily of acetyltransferases. Arch Biochem Biophys.

[38] Strahilevitz J, Jacoby GA, Hooper DC, Robicsek A (2009). Plasmid-mediated quinolone resistance: a multifaceted threat. Clin Microbiol Rev.

[39] Coque TM, Singh KV, Weinstock GM, Murray BE (1999). Characterization of dihydrofolate reductase genes from trimethoprim-susceptible and trimethoprim-resistant strains of *Enterococcus faecalis*. Antimicrob Agents Chemother.

[40] van Hoek A, Mevius D, Guerra B, Mullany P, Roberts A, Aarts H (2011). Acquired antibiotic resistance genes: an overview. Front Microbiol.

[41] Meziane-Cherif D, Stogios PJ, Evdokimova E, Savchenko A, Courvalin P (2014). Structural basis for the evolution of vancomycin resistance D,D-peptidases. Proc Natl Acad Sci U S A.

[42] Oliveira H, Melo LDR, Santos SB, Nóbrega FL, Ferreira EC, Cerca N (2013). Molecular aspects and comparative genomics of bacteriophage endolysins. J Virol.

[43] Ambler RP, Coulson AF, Frère JM, Ghuysen JM, Joris B, Forsman M (1991). A standard numbering scheme for the class A β-lactamases. Biochem J.

[44] Callebaut I, Moshous D, Mornon J-P, de Villartay J-P (2002). Metallo-β-lactamase fold within nucleic acids processing enzymes: the β-CASP family. Nucleic Acids Res.

[45] Delbruck H, Bogaerts P, Kupper MB (2012). Rezende de Castro R, Bennink S, Glupczynski Y, et al. Kinetic and crystallographic studies of extended-spectrum GES-11, GES-12, and GES-14 β-lactamases. Antimicrob Agents Chemother.

[46] Lee JH, Takahashi M, Jeon JH, Kang LW, Seki M, Park KS (2019). Dual activity of PNGM-1 pinpoints the evolutionary origin of subclass B3 metallo-β-lactamases: a molecular and evolutionary study. Emerg Microbes Infect.

[47] Stoczko M, Frere JM, Rossolini GM, Docquier JD (2008). Functional diversity among metallo-β-lactamases: characterization of the CAR-1 enzyme of *Erwinia carotovora*. Antimicrob Agents Chemother.

[48] Ren J, Song K, Deng C, Ahlgren NA, Fuhrman JA, Li Y, et al. Identifying viruses from metagenomic data using deep learning. Quant Biol. 2020. 10.1007/s40484-019-0187-4 PMC8172088 34084563

[49] Williamson SJ, Rusch DB, Yooseph S, Halpern AL, Heidelberg KB, Glass JI (2008). The Sorcerer II global ocean sampling expedition: metagenomic characterization of viruses within aquatic microbial samples. PLoS One.

[50] Balcazar JL (2014). Bacteriophages as vehicles for antibiotic resistance genes in the environment. PLoS Pathog.

[51] Li B, Yang Y, Ma L, Ju F, Guo F, Tiedje JM (2015). Metagenomic and network analysis reveal wide distribution and co-occurrence of environmental antibiotic resistance genes. ISME J.

[52] Chao Y, Ma L, Yang Y, Ju F, Zhang X-X, Wu W-M (2013). Metagenomic analysis reveals significant changes of microbial compositions and protective functions during drinking water treatment. Sci Rep.

[53] Jeon JH, Lee JH, Lee JJ, Park KS, Karim AM, Lee CR (2015). Structural basis for carbapenem-hydrolyzing mechanisms of carbapenemases conferring antibiotic resistance. Int J Mol Sci.

[54] Medeiros AA (1997). Evolution and dissemination of β-lactamases accelerated by generations of β-lactam antibiotics. Clin Infect Dis.

[55] Elbehery AH, Leak DJ, Siam R (2017). Novel thermostable antibiotic resistance enzymes from the Atlantis II Deep Red Sea brine pool. Microb Biotechnol.

[56] Kubota H, Suzuki Y, Okuno R, Uchitani Y, Ariyoshi T, Takemura N (2019). IMP-68, a Novel IMP-type metallo-β-lactamase in imipenem-susceptible *Klebsiella pneumoniae*. mSphere.

[57] Ur Rahman S, Ali T, Ali I, Khan NA, Han B, Gao J (2018). The growing genetic and functional diversity of extended spectrum β-lactamases. Biomed Res Int.

[58] Calero-Cáceres W, Muniesa M (2016). Persistence of naturally occurring antibiotic resistance genes in the bacteria and bacteriophage fractions of wastewater. Water Res.

[59] Coutinho FH, Gregoracci GB, Walter JM, Thompson CC, Thompson FL (2018). Metagenomics sheds light on the ecology of marine microbes and their viruses. Trends Microbiol.

[60] Pehrsson E, Forsberg K, Gibson M, Ahmadi S, Dantas G (2013). Novel resistance functions uncovered using functional metagenomic investigations of resistance reservoirs. Front Microbiol.

[61] Marathe NP, Janzon A, Kotsakis SD, Flach C-F, Razavi M, Berglund F (2018). Functional metagenomics reveals a novel carbapenem-hydrolyzing mobile β-lactamase from Indian river sediments contaminated with antibiotic production waste. Environ Int.

[62] Boulund F, Berglund F, Flach C-F, Bengtsson-Palme J, Marathe NP, Larsson DGJ (2017). Computational discovery and functional validation of novel fluoroquinolone resistance genes in public metagenomic data sets. BMC Genomics.

[63] Kupczok A, Neve H, Huang KD, Heoppner MP, Heller KJ, Franz CMAP (2018). Rates of mutations and recombination is *Siphoviridae* phage genome evolution over three decades. Mol Biol Evol.

[64] Sanjuán R, Nebot MR, Chirico N, Mansky LM, Belshaw R (2010). Viral mutation rates. J Virol.

[65] Hevroni G, Enav H, Rohwer F, Beja O (2015). Diversity of viral photosystem-I psaA genes. ISME J.

[66] John SG, Mendez CB, Deng L, Poulos B, Kauffman AK, Kern S (2011). A simple and efficient method for concentration of ocean viruses by chemical flocculation. Environ Microbiol Rep.

[67] Hurwitz BL, Deng L, Poulos BT, Sullivan MB (2013). Evaluation of methods to concentrate and purify ocean virus communities through comparative, replicated metagenomics. Environ Microbiol.

[68] Thurber RV, Haynes M, Breitbart M, Wegley L, Rohwer F (2009). Laboratory procedures to generate viral metagenomes. Nat Protoc.

[69] Bolger AM, Lohse M, Usadel B (2014). Trimmomatic: a flexible trimmer for Illumina sequence data. Bioinformatics..

[70] Wilke A, Harrison T, Wilkening J, Field D, Glass EM, Kyrpides N (2012). The M5nr: a novel non-redundant database containing protein sequences and annotations from multiple sources and associated tools. BMC Bioinformatics.

[71] Bengtsson-Palme J, Hartmann M, Eriksson KM, Pal C, Thorell K, Larsson DGJ (2015). METAXA2: improved identification and taxonomic classification of small and large subunit rRNA in metagenomic data. Mol Ecol Resour.

[72] Zolfo M, Pinto F, Asnicar F, Manghi P, Tett A, Bushman FD (2019). Detecting contamination in viromes using ViromeQC. Nat Biotechnol.

[73] Bankevich A, Nurk S, Antipov D, Gurevich AA, Dvorkin M, Kulikov AS (2012). SPAdes: a new genome assembly algorithm and its applications to single-cell sequencing. J Comput Biol.

[74] Roux S, Enault F, Hurwitz BL, Sullivan MB (2015). VirSorter: mining viral signal from microbial genomic data. PeerJ..

[75] Lavigne R, Seto D, Mahadevan P, Ackermann H-W, Kropinski AM (2008). Unifying classical and molecular taxonomic classification: analysis of the Podoviridae using BLASTP-based tools. Res Microbiol.

[76] Söding J (2005). Protein homology detection by HMM–HMM comparison. Bioinformatics..

[77] Sievers F, Wilm A, Dineen D, Gibson TJ, Karplus K, Li W (2011). Fast, scalable generation of high-quality protein multiple sequence alignments using Clustal Omega. Mol Syst Biol.

[78] Kumar S, Stecher G, Tamura K (2016). MEGA7: Molecular Evolutionary Genetics Analysis version 7.0 for bigger datasets. Mol Biol Evol.

[79] Patel JB, Cockerill FR, Bradford PA, Eliopoulos GM, Hindler JA, Jenkins SG (2015). Methods for dilution antimicrobial susceptibility tests for bacteria that grow aerobically; approved standard-tenth edition.

[80] Kuzmic P (1996). Program DYNAFIT for the analysis of enzyme kinetic data: application to HIV proteinase. Anal Biochem.

[81] Lo CC, Chain PS (2014). Rapid evaluation and quality control of next generation sequencing data with FaQCs. BMC Bioinformatics.

[82] Peng Y, Leung HC, Yiu SM, Chin FY (2012). IDBA-UD: a de novo assembler for single-cell and metagenomic sequencing data with highly uneven depth. Bioinformatics..

[83] Hyatt D, Chen G-L, LoCascio PF, Land ML, Larimer FW, Hauser LJ (2010). Prodigal: prokaryotic gene recognition and translation initiation site identification. BMC Bioinformatics.

