# Supplementary material for: Freshwater viral metagenome reveals novel and functional phage-borne antibiotic resistance genes
Source: Microbiome. 2020 Jun 1;8:75. doi: 10.1186/s40168-020-00863-4 (PMC7265639; doi:10.1186/s40168-020-00863-4)
Supplement: Supplementary file 2 — Additional file 1: Tables S1-S10 and FiguresS1-S14. [file 40168_2020_863_MOESM1_ESM.zip › Resubmission_Microbiome_ARG_supplementary_REV6.docx]

Previous Manuscript ID: MBIO-D-19-01077

Supplementary Information for

Freshwater Viral Metagenome Reveals Novel and Functional Phage-borne Antibiotic Resistance Genes

Kira Moon^1^, Jeong Ho Jeon^2^, Ilnam Kang^1^, Kwang Seung Park^2^, Kihyun Lee^3^, Chang-Jun Cha^3^, Sang Hee Lee^2*^ and Jang-Cheon Cho^1*^

^1^ Department of Biological Sciences, Inha University, Incheon 22212, Republic of Korea

^2^ National Leading Research Laboratory of Drug Resistance Proteomics, Department of Biological Sciences, Myongji University, 116 Myongjiro, Yongin, Gyeonggi-do 17058, Republic of Korea

^3^ Department of Systems Biotechnology and Center for Antibiotic Resistome, Chung-Ang University, Anseong, Gyeonggi-do 17546, Republic of Korea

*Correspondence: [chojc@inha.ac.kr](mailto:chojc@inha.ac.kr); [sangheelee@mju.ac.kr](mailto:sangheelee@mju.ac.kr)

**This file includes:**

**Table S1**. Numbers of reads, viral contigs, ORFs, and β-lactamases in the viral contigs from the Han River viral metagenome datasets

**Table S2**. Percentage of bacterial gene markers detected within the Han River virome datasets

**Table S3.** List of representative class A, B, C, and D β-lactamases used to construct phylogenetic trees for HRV-1 and HRVM-1 (Provided in a separate Excel file)

**Table S4.** Minimum inhibitory concentrations (MICs) of diverse β-lactams for *Escherichia coli* BL21 (DE3) transformants expressing HRV-1 or HRVM-1

**Table S5.** Kinetic parameters of HRV-1 and HRVM-1 β-lactamases for various β-lactams

**Table S6.** List of BLASTp match results of HRV-1 against different public databases and Han River bacterial metagenome contigs (Provided in a separate Excel file)

**Table S7.** List of BLASTp match results of HRVM-1 against different public databases and the Han River bacterial metagenome contigs (Provided in a separate Excel file)

**Table S8**. Number of ARGs detected within viral contig ORFs using different thresholds

**Table S9.** Strains, plasmids, and primers used in this study

**Table S10.** Characteristic molecular extinction coefficients of substrates used in this study

**Figure S1.** Map of the Han River showing the six sampling stations marked with triangles

**Figure S2.** Functional gene annotation of viral metagenome reads collected from six sites on the Han River

**Figure S3.** Taxonomic distribution of the Han River viral metagenome reads assigned to bacterial taxonomy at the class level with bacteriophage functions

**Figure S4.** Sequence maps of viral contigs carrying antibiotic resistance genes *vatA* and v*atB*

**Figure S5.** Sequence maps of viral contigs carrying acetyltransferase genes that confer antibiotic resistance

**Figure S6.** Sequence maps of viral contigs carrying the antibiotic resistance gene *dfrB2*

**Figure S7.** Sequence maps of viral contigs carrying *qnr* or *van* antibiotic resistance genes

**Figure S8.** Amino acid sequence alignment of the *vatB* gene carried by the viral contig N1-C442-G2 with representative VatA and VatB sequences from diverse bacterial strains

**Figure S9.** Amino acid sequences of AAC(6’) found in viral contigs from the Han River virome

**Figure S10.** Amino acid sequence alignment of H4-C441-ORF28 with representative sequences of class A β-lactamases

**Figure S11.** Amino acid sequence alignment of the three ORFs (H1-C74-ORF21, H4-C244-ORF21, and H4-C367-ORF18) with representative sequences of subclass B3 β-lactamases

**Figure S12.** Amino acid sequence alignments of HRV-1 and HRVM-1 with highly related sequences discovered from the bacterial and viral metagenomes

**Figure S13.** Viral proteomic tree of Han River viral contigs harboring ARG sequences constructed with the ViPTree program

**Figure S14.** Gene-sharing network of Han River viral contigs harboring ARG sequences constructed with the vConTACT v.2.0 program

**Table S1**. Numbers of reads, viral contigs, and ORFs in the viral contigs from the Han River virome datasets

| Site | No. of reads^a^ | Annotated reads (%) | No. of contigs | No. of contigs  (≥ 10-kbp) | No. of viral contigs  (≥10-kbp)^b^ | No. of ORFs |
| --- | --- | --- | --- | --- | --- | --- |
| N1 | 4,739,263 | 77.3 | 93,140 | 976 | 938 | 27,948 |
| N3 | 4,253,428 | 80.7 | 113,682 | 610 | 588 | 16,951 |
| H1 | 3,649,892 | 87.9 | 275,322 | 760 | 725 | 19,638 |
| H3 | 6,006,517 | 84.0 | 280,209 | 1,420 | 1,361 | 38,107 |
| H4 | 6,630,128 | 77.6 | 248,253 | 982 | 936 | 25,633 |
| H6 | 4,818,906 | 83.4 | 328,339 | 787 | 747 | 20,424 |
| Total |  |  | 1,338,945 | 5,535 | 5,295 | 148,701 |

^a^Number of viral metagenome reads after quality trimming using Trimmomatic program.

^b^Metagenome contigs were predicted to be viral if a contig contained one or more virus-related genes, according to VirSorter program.

| Site | METAXA |  | ViromeQC | | |
| --- | --- | --- | --- | --- | --- |
|  | 16S rRNA seq.^a^ |  | SSU rRNA seq. | LSU rRNA seq. | Bacterial marker seq.^b^ |
| N1 | 0.00025% |  | 0.00112% | 0.02122% | 0.00918% |
| N3 | 0.00007% |  | 0.00115% | 0.00685% | 0.00955% |
| H1 | 0.00030% |  | 0.00133% | 0.00541% | 0.01565% |
| H3 | 0.00030% |  | 0.00132% | 0.00660% | 0.01568% |
| H4 | 0.00009% |  | 0.00102% | 0.00381% | 0.01180% |
| H6 | 0.00013% |  | 0.00093% | 0.00344% | 0.01081% |

**Table S2**. Percentage of bacterial gene markers detected within the Han River virome datasets

^a^Percent of both SSU and LSU rRNA sequences detected within the virome reads using the METAXA program

^b^Percent of single-copy bacterial marker gene sequences detected within the virome reads using the Virome QC program.

**Table S3. (Provided in a separate excel file)**

**Table S4.** Minimum inhibitory concentrations (MICs) of diverse β-lactams for *Escherichia coli* BL21 (DE3) transformants expressing HRV-1 or HRVM-1

| β-Lactam | MIC (μg/mL) for *E. coli* strain | | |
| --- | --- | --- | --- |
|  | *E. coli* BL21 (DE3)-pET-28a(+)/ *E. coli* BL21 (DE3)-pET-30a(+) | *E. coli* BL21 (DE3)-pET-30a(+)-HRV-1 | *E. coli* BL21 (DE3)-pET-28a(+)-HRVM-1 |
| Benzylpenicillin | 32/32 | 512 | 256 |
| Ampicillin | 4/4 | 64 | 32 |
| Piperacillin | 2/2 | 64 | 32 |
| Ticarcillin | 4/4 | 32 | 32 |
| Oxacillin | 4/4 | 256 | 128 |
| Cloxacillin | 2/2 | 256 | 128 |
| Cephalothin | 8/8 | 32 | 32 |
| Cefoxitin | 0.25/0.25 | 4 | 4 |
| Cefotaxime | 0.125/0.125 | 8 | 8 |
| Ceftazidime | 0.5/0.5 | 8 | 8 |
| Cefepime | 0.06/0.06 | 4 | 4 |
| Aztreonam | 0.06/0.06 | 8 | 8 |
| Imipenem | 0.06/0.06 | 0.5 | 0.5 |
| Meropenem | 0.06/0.06 | 0.5 | 0.5 |
| Ertapenem | 0.06/0.06 | 0.5 | 0.5 |

**Table S5.** Kinetic parameters of HRV-1 and HRVM-1 β-lactamases for various β-lactams

| Substrate | *K*_m_ (μM) | | *k*_cat_ (s^–1^) | | *k*_cat_/*K*_m_ (M^–1^ s^–1^) | |
| --- | --- | --- | --- | --- | --- | --- |
|  | HRV-1 | HRVM-1 | HRV-1 | HRVM-1 | HRV-1 | HRVM-1 |
| Benzylpenicillin | 11.53 ± 0.01 | 10.84 ± 0.01 | 12.34 ± 0.01 | 8.91 ± 0.01 | (1.07 ± 0.01) × 10^6^ | (8.2 ± 0.01) × 10^5^ |
| Ampicillin | 8.82 ± 0.01 | 8.93 ± 0.01 | 6.09 ± 0.01 | 5.72 ± 0.01 | (6.9 ± 0.01) × 10^5^ | (6.4 ± 0.01) × 10^5^ |
| Piperacillin | 16.81 ± 0.01 | 16.14 ± 0.01 | 0.83 ± 0.01 | 0.81 ± 0.01 | (5.0 ± 0.01) × 10^4^ | (5.0 ± 0.01) × 10^4^ |
| Ticarcillin | 16.74 ± 0.01 | 16.74 ± 0.01 | 0.67 ± 0.01 | 0.50 ± 0.01 | (4.0 ± 0.01) × 10^4^ | (3.0 ± 0.01) × 10^4^ |
| Oxacillin | 13.86 ± 0.01 | 12.08 ± 0.01 | 0.65 ± 0.01 | 0.43 ± 0.01 | (4.7 ± 0.01) × 10^4^ | (3.6 ± 0.01) × 10^4^ |
| Cloxacillin | 11.29 ± 0.01 | 12.87 ± 0.01 | 0.61 ± 0.01 | 0.57 ± 0.01 | (5.4 ± 0.01) × 10^4^ | (4.4 ± 0.01) × 10^4^ |
| Cephalothin | 13.17 ± 0.01 | 12.07 ± 0.01 | 0.49 ± 0.01 | 0.41 ± 0.01 | (3.8 ± 0.01) × 10^4^ | (3.4 ± 0.01) × 10^4^ |
| Cefoxitin | 14.92 ± 0.02 | 13.54 ± 0.01 | 0.026 ± 0.02 | 0.015 ± 0.01 | (1.7 ± 0.02) × 10^3^ | (1.1 ± 0.01) × 10^3^ |
| Cefotaxime | 11.74 ± 0.01 | 11.83 ± 0.03 | 0.048 ± 0.01 | 0.01 ± 0.03 | (4.1 ± 0.01) × 10^3^ | (9.0 ± 0.03) × 10^2^ |
| Ceftazidime | 12.15 ± 0.02 | 12.03 ± 0.02 | 0.041 ± 0.02 | 0.01 ± 0.02 | (3.3 ± 0.02) × 10^3^ | (8.0 ± 0.02) × 10^2^ |
| Cefepime | 12.00 ± 0.01 | 10.48 ± 0.01 | 0.012 ± 0.01 | 0.01 ± 0.01 | (1.0 ± 0.01) × 10^3^ | (9.0 ± 0.01) × 10^2^ |
| Aztreonam | 18.68 ± 0.01 | 18.17 ± 0.01 | 0.0309 ± 0.01 | 0.0258 ± 0.01 | (1.7 ± 0.01) × 10^3^ | (1.4 ± 0.01) × 10^3^ |
| Imipenem | 6.17 ± 0.01 | 5.42 ± 0.01 | 0.0051 ± 0.01 | 0.0026 ± 0.01 | (8.0 ± 0.01) × 10^2^ | (5.0 ± 0.01) × 10^2^ |
| Meropenem | 8.46 ± 0.01 | 8.80 ± 0.01 | 0.0039 ± 0.01 | 0.0018 ± 0.01 | (5.0 ± 0.01) × 10^2^ | (2.0 ± 0.01) × 10^2^ |
| Ertapenem | 8.07 ± 0.01 | 8.36 ± 0.01 | 0.0045 ± 0.01 | 0.0021 ± 0.01 | (6.0 ± 0.01) × 10^2^ | (3.0 ± 0.01) × 10^2^ |

**Tables S6 & S7. (Provided in a separate excel file)**

**Table S8**. Number of ARGs detected within viral contig ORFs using different thresholds

| Thresholds | Reference | N1 | N3 | H1 | H4 | H6 | H7 |
| --- | --- | --- | --- | --- | --- | --- | --- |
| AA % ident. ≥ 80%,  Seq. cov. ≥ 85% | Enault *et al*., 2017, Gibson *et al*., 2015 | 0 | 0 | 0 | 0 | 0 | 0 |
| AA % ident. ≥ 80%,  Seq. cov. ≥ 25 AA | Lekunberri *et al*., 2017 | 0 | 0 | 0 | 0 | 0 | 0 |
| AA % ident. ≥ 90%,  Seq. cov. ≥ 90% | Subirats *et al*., 2016 | 0 | 0 | 0 | 0 | 0 | 0 |
| AA % ident. ≥ 90%,  Seq. cov. ≥ 25,  e-value ≤ 10^-5^ | Li *et al*., 2015 | 0 | 0 | 0 | 0 | 0 | 0 |
| AA % ident. ≥ 90%,  Seq. cov. ≥ 25,  e-value ≤ 10^-6^ | Chao *et al*., 2013 | 0 | 0 | 0 | 0 | 0 | 0 |

**Table S9.** Strains, plasmids, and primers used in this study

| Strains, plasmids, and primers | Phenotype, genotype and/or characteristics | Source  (or reference) |
| --- | --- | --- |
| Strains |  |  |
| *E. coli* BL21(DE3) | F^-^*ompT hsdS_B_*(r_B_^-^m_B_^-^) *gal dcm* (DE3) | Invitrogen |
| Plasmids |  |  |
| pET-28a(+) | Expression vector, kanamycin^r^ | Novagen |
| pET-30a(+) | Expression vector, kanamycin^r^ | Novagen |
| pET-30a(+)/*bla*_HRV-1_-His_6_ | pET-30a(+) containing *bla*_HRV-1_ without signal peptide from the Han River virome | This study |
| pET-28a(+)/*bla*_HRVM-1_-His_6_ | pET-28a(+) containing *bla*_HRVM-1_ from the Han River virome | This study |
| Primers |  |  |
| *Nde*I-HRV-1-F | 5'–ATA **CATATG** AATACTACATACGTTTACAATGTAACTAAAGAACAGG–3' | This study |
| *Xho*I-EK-HRV-1-R | 5'–GAG **CTCGAG** GTCGTCGTCGTCCTT AATTGCTTCATTTAAAATTTTTCG TGCGGTCTTTTG–3' | This study |
| *Nco*I-HRVM-1-F | 5'–ATA **CCATGG** GC ATGGAATTAAAAGTAATTTCATCTGGAAGTATTGGC–3' | This study |
| *Xho*I-EK-HRVM-1-R | 5'–GAG **CTCGAG** GTCGTCGTCGTCCTT AAAATTTTCTAGGTTAATAAT TTG TCCTTTTGTGGCAAT–3' | This study |

Restriction sites appear in bold. The underlined bases indicate the enterokinase recognition site. r: resistant.

**Table S10.** Characteristic molecular extinction coefficients of substrates used in this study

| Substrate | Absorbance | Molecular extinction coefficient |
| --- | --- | --- |
| Benzylpenicillin | Δε_233nm_ | −780 M^−1^ cm^−1^ |
| Ampicillin | Δε_235nm_ | −900 M^−1^ cm^−1^ |
| Piperacillin | Δε_235nm_ | −793 M^−1^ cm^−1^ |
| Ticarcillin | Δε_235nm_ | −900 M^−1^ cm^−1^ |
| Oxacillin | Δε_260nm_ | −1 000 M^−1^ cm^−1^ |
| Cloxacillin | Δε_260nm_ | −1 000 M^−1^ cm^−1^ |
| Cephalothin | Δε_262nm_ | −7 660 M^−1^ cm^−1^ |
| Cefoxitin | Δε_270nm_ | −8 380 M^−1^ cm^−1^ |
| Cefotaxime | Δε_264nm_ | −7 250 M^−1^ cm^−1^ |
| Ceftazidime | Δε_265nm_ | −10 300 M^−1^ cm^−1^ |
| Aztreonam | Δε_318nm_ | −650 M^−1^ cm^−1^ |
| Imipenem | Δε_278nm_ | −5 660 M^−1^ cm^−1^ |
| Meropenem | Δε_298nm_ | −9 530 M^−1^ cm^−1^ |
| Ertapenem | Δε_295nm_ | −10 940 M^−1^ cm^−1^ |


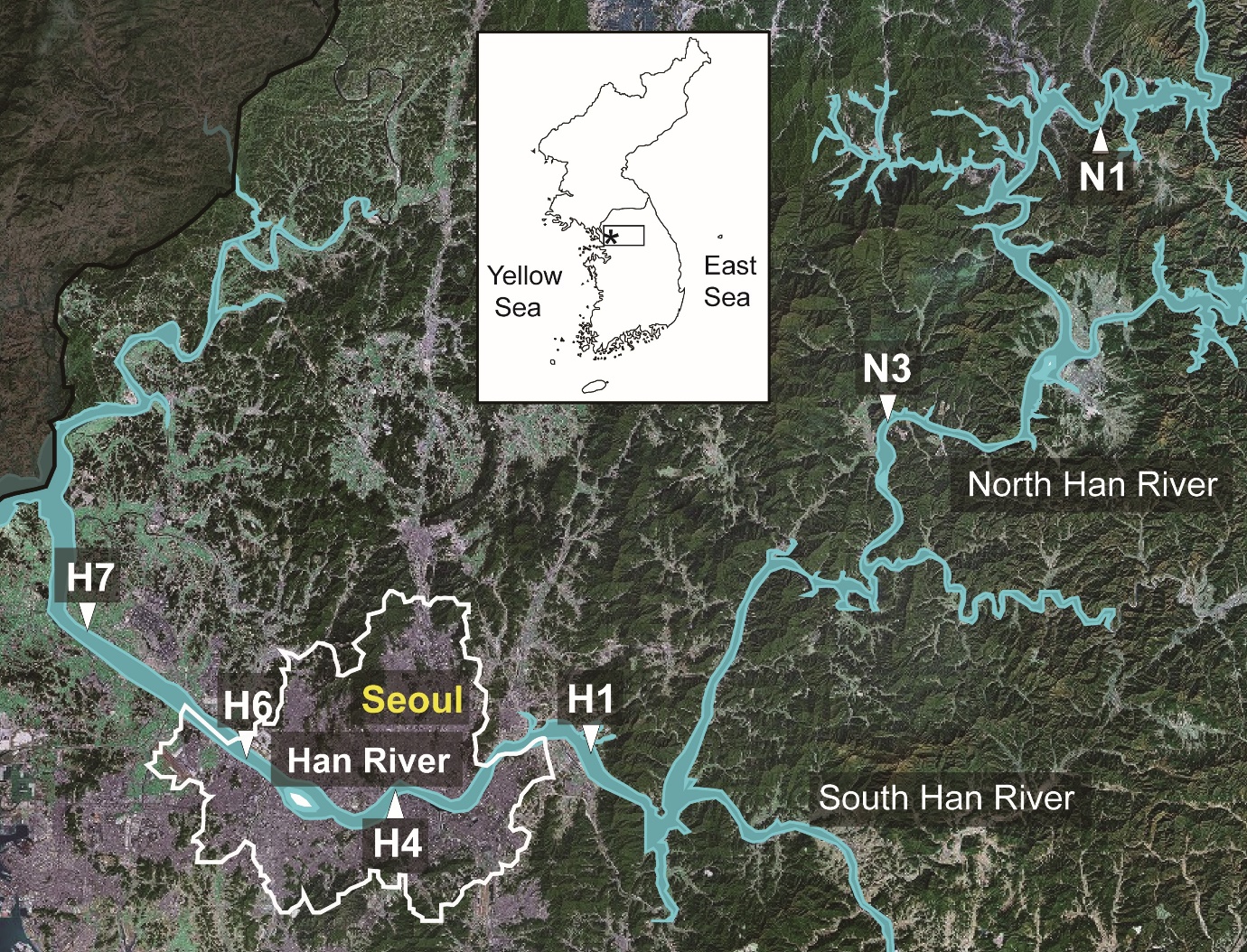


**Figure S1.** Map of the Han River showing the six sampling stations marked with triangles


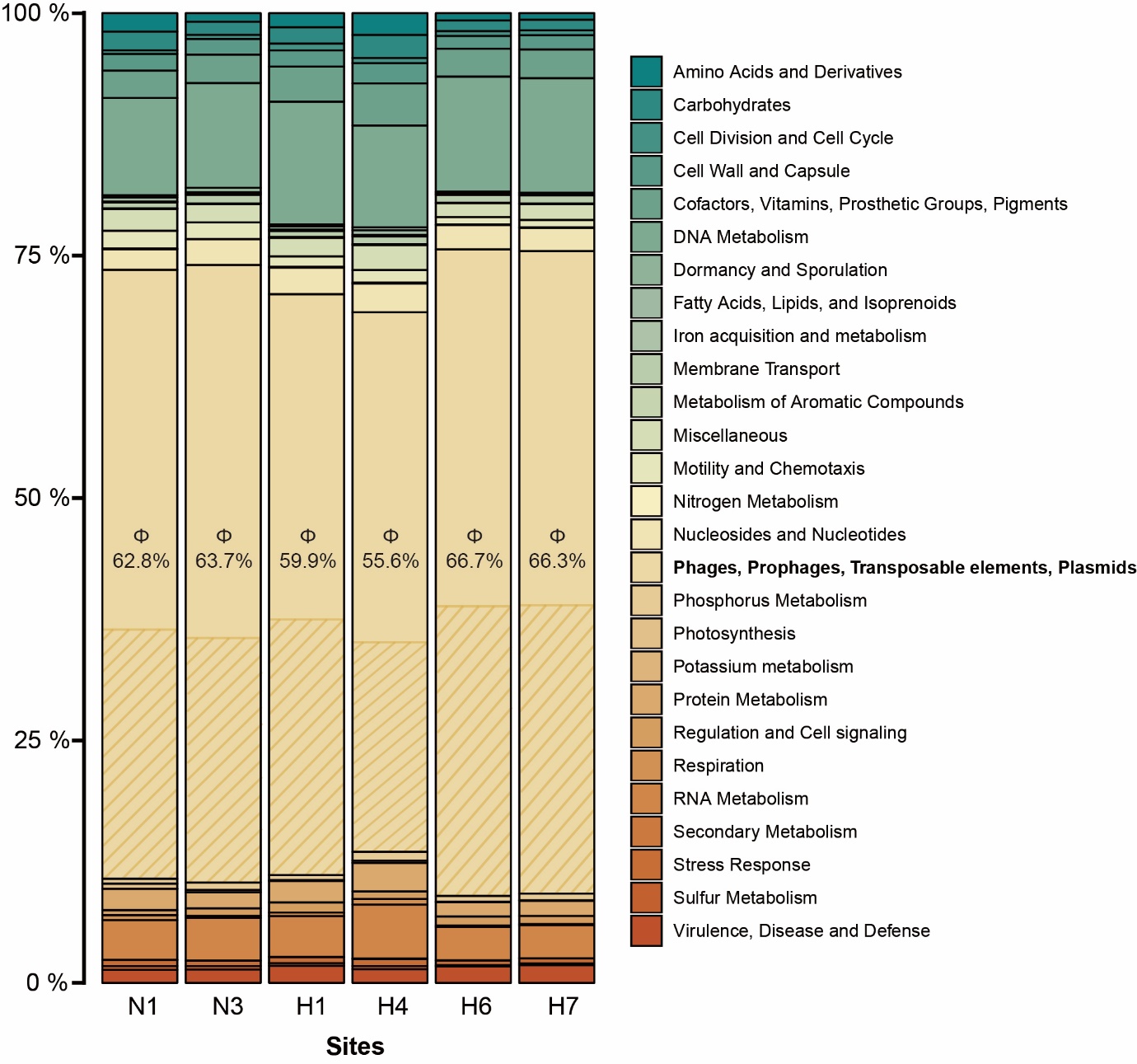


**Figure S2.** Functional gene annotation of viral metagenome reads collected from six sites on the Han River. The shaded area within the “Phages, prophages, Transposable elements, Plasmids” criteria indicates those taxonomically annotated as bacteria.


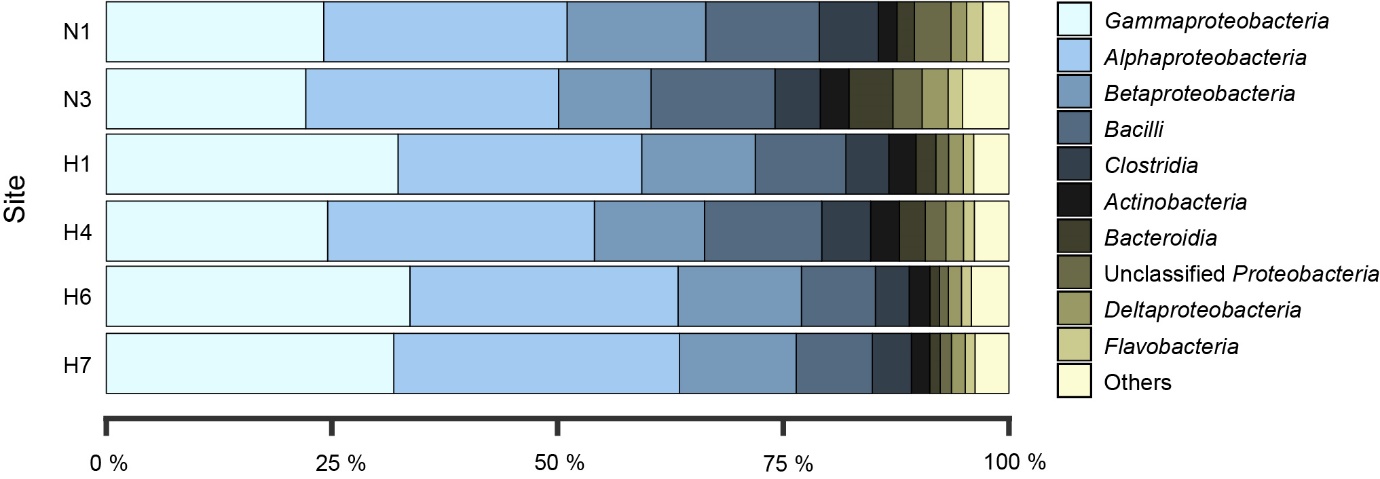


**Figure S3.** Taxonomic distribution of the Han River viral metagenome reads assigned to bacterial taxonomy at the class level with bacteriophage functions (5.61-7.82% of total assigned reads)


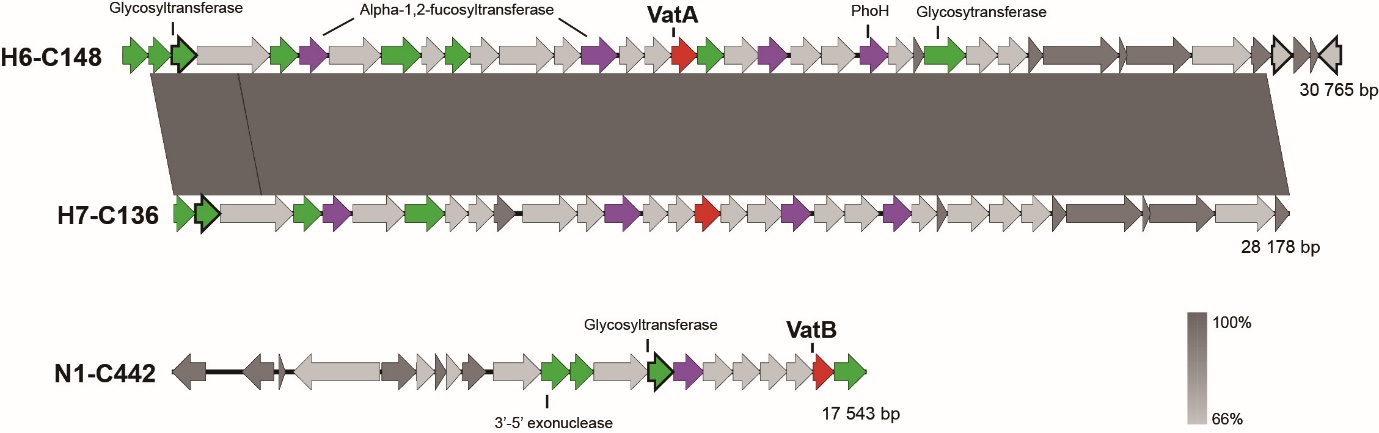


**Figure S4.** Sequence maps of viral contigs carrying antibiotic resistance genes *vatA* and v*atB*, shown in red. The green represents DNA replication, recombination, and modification-related genes, purple represents auxiliary metabolic genes while light grey represents hypothesis genes and dark grey genes unannotated genes. The bold-lined arrows indicate that they were originated from a virus.


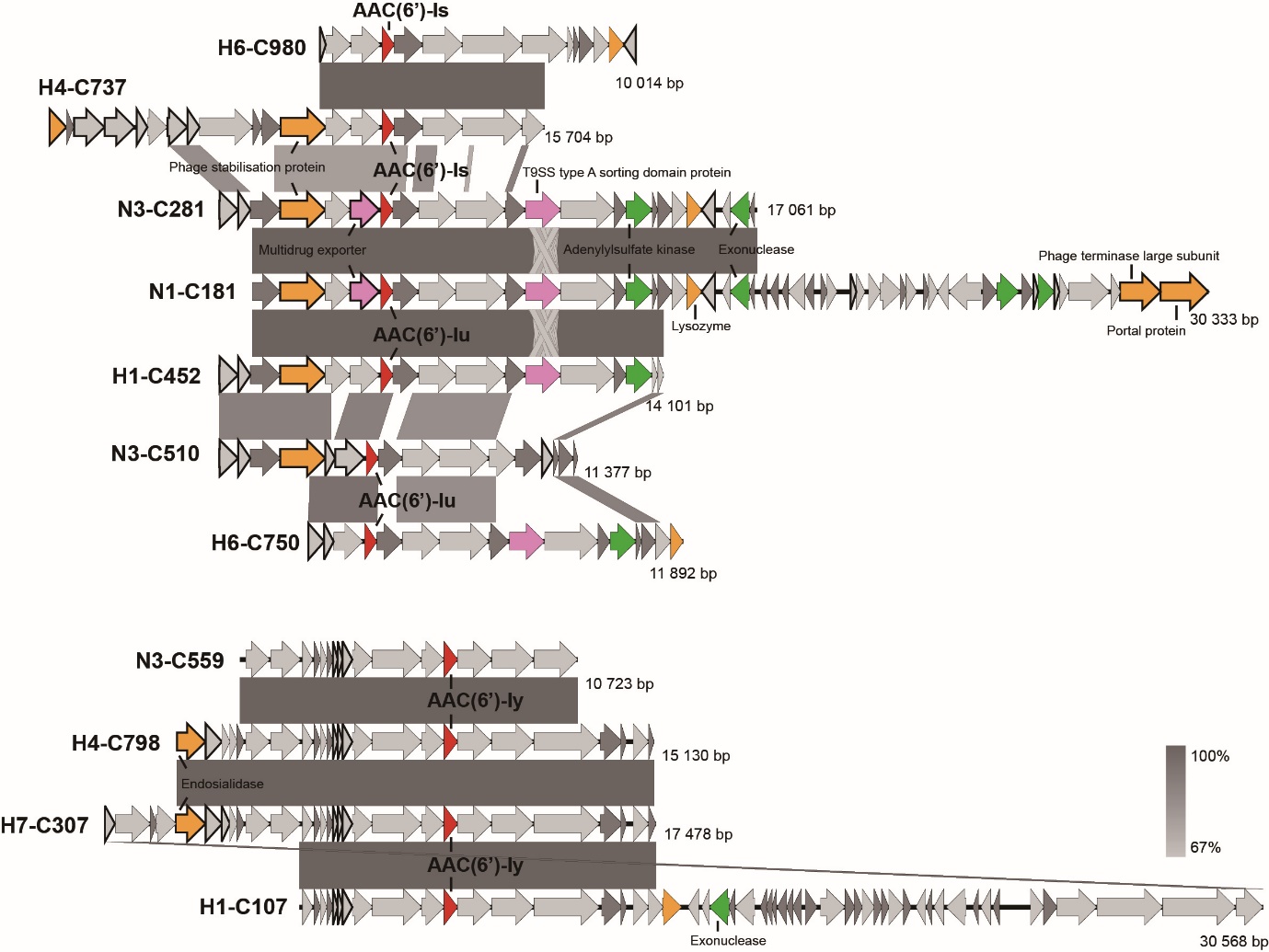


**Figure S5.** Sequence maps of viral contigs carrying acetyltransferase genes that confer antibiotic resistance. The green represents DNA replication, recombination, and modification-related genes, yellow indicates cell lysis and packaging genes, pink represents antibiotic resistance genes other than acetyltransferase genes while light grey represents hypothesis genes and dark grey genes unannotated genes. The bold-lined arrows indicate that they were originated from a virus.


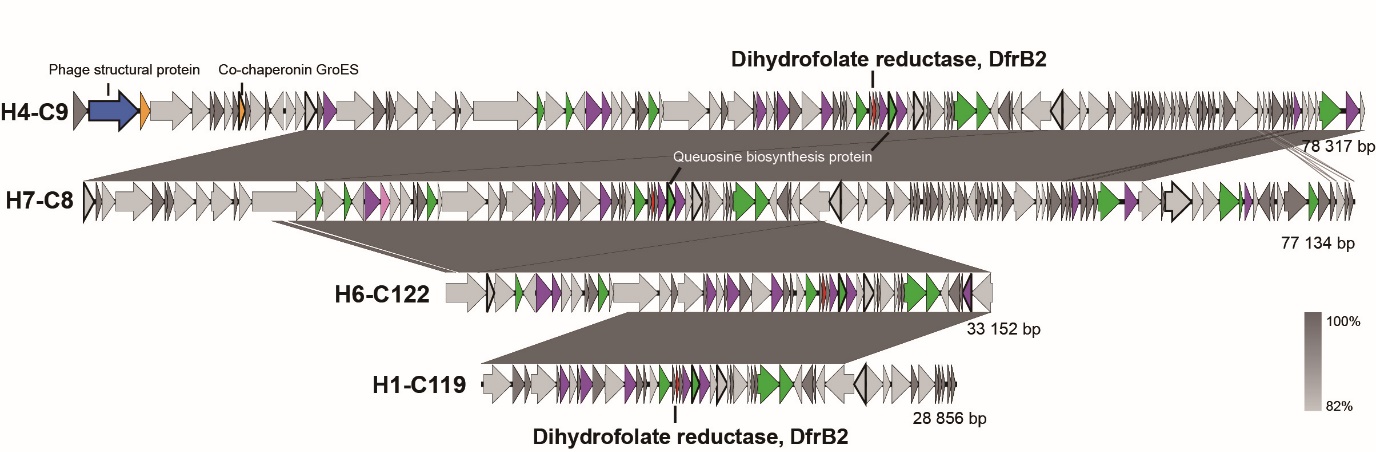


**Figure S6.** Sequence maps of viral contigs carrying the antibiotic resistance gene *dfrB2*, shown in red. The blue indicates structural genes, green represents DNA replication, recombination, and modification-related genes, yellow indicates cell lysis and packaging genes, purple represents auxiliary metabolic genes while light grey represents hypothesis genes and dark grey genes unannotated genes. The bold-lined arrows indicate that they were originated from a virus.


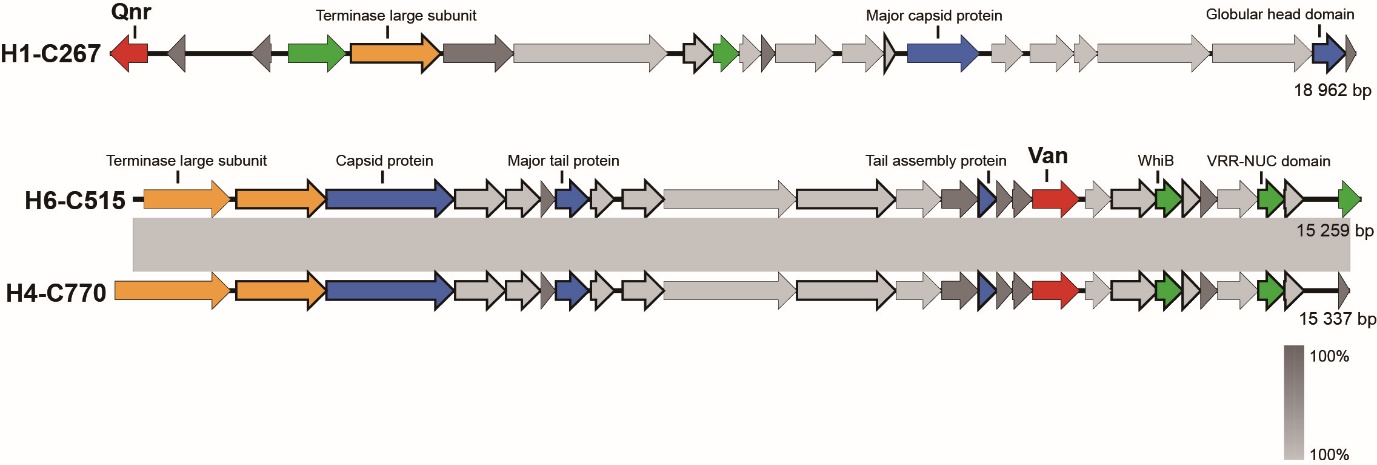


**Figure S7.** Sequence maps of viral contigs carrying *qnr* or *van* antibiotic resistance genes, which are shown in red. The blue indicates structural genes, green represents DNA replication, recombination, and modification-related genes, yellow indicates cell lysis and packaging genes, while light grey represents hypothesis genes and dark grey genes unannotated genes. The bold-lined arrows indicate that they were originated from a virus.


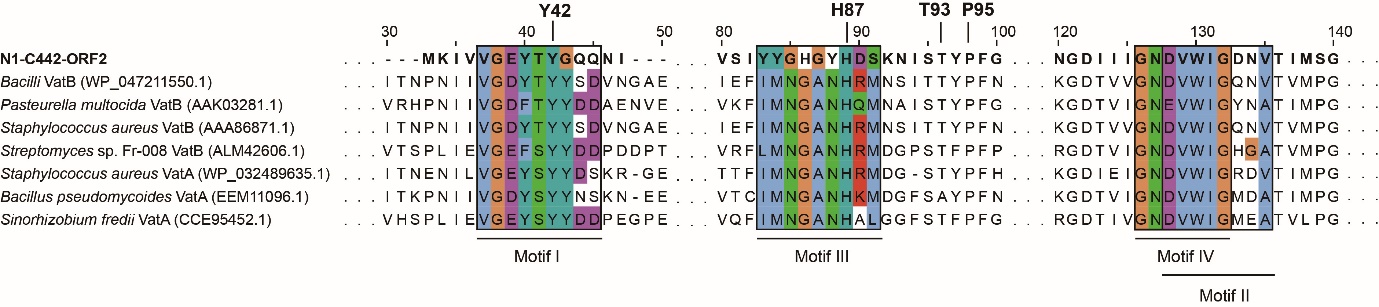


**Figure S8.** Amino acid sequence alignment of the *vatB* gene carried by the viral contig N1-C442-G2 with representative VatA and VatB sequences from diverse bacterial strains. The sequences were aligned using the ClustalO program. Highly conserved enzyme motifs I to IV of Vat gene family are marked with colors.


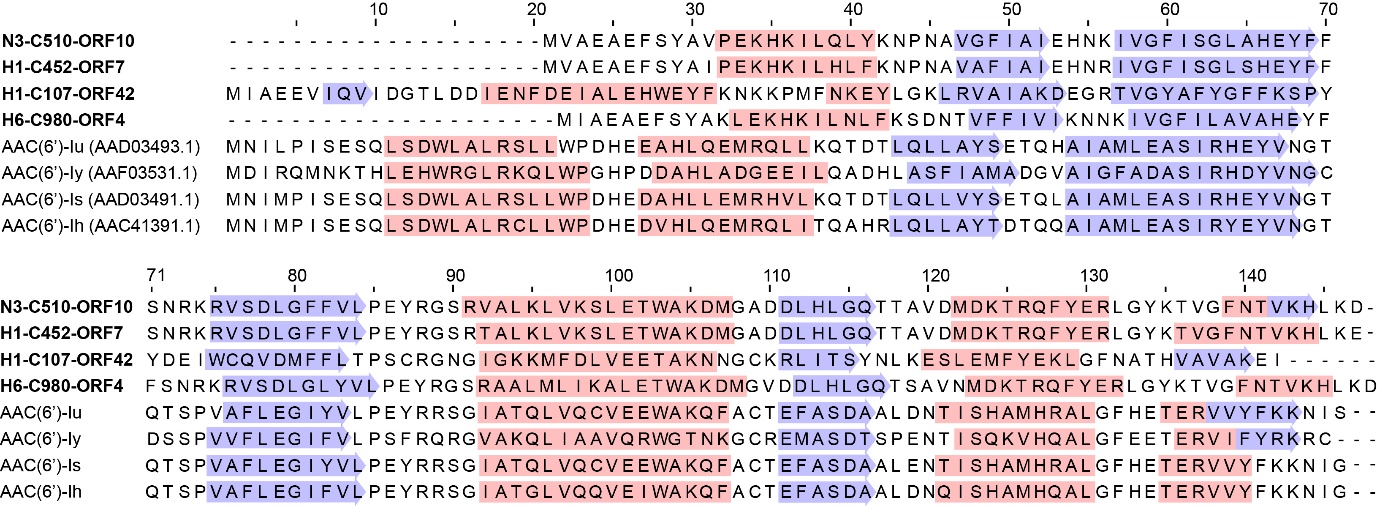


**Figure S9.** Amino acid sequences of AAC(6’) found in viral contigs from the Han River virome. The blue ribbon represents beta-sheet structure while the pink ribbon represents the alpha-sheet structure. The sequences are manually arranged to visualize the patterns of the predicted secondary structure of the genes.


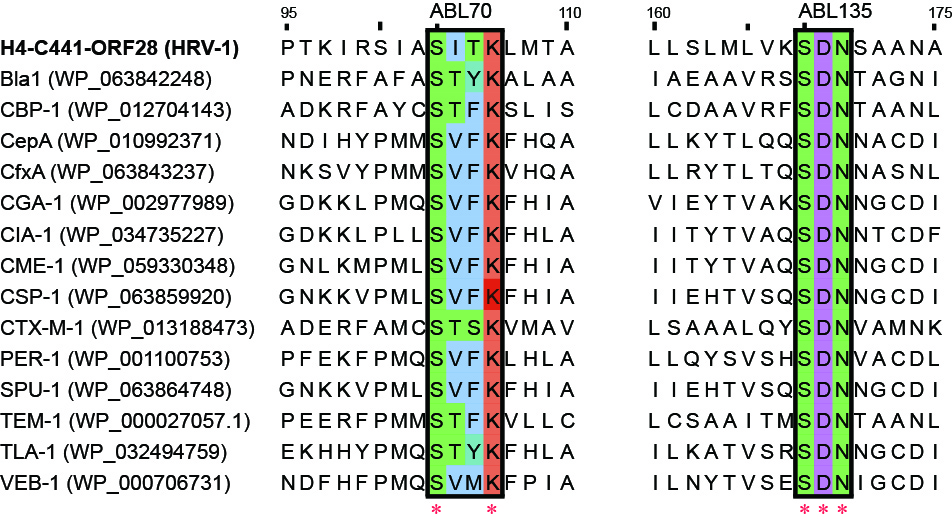


**Figure S10.** Amino acid sequence alignment of H4-C441-ORF28 with representative sequences of class A β-lactamases. H4-C441-ORF28, retrieved from the Han River viromes harbors conserved active sites of class A β-lactamases, which are “S-X-X-K” and “S-D-N” and was therefore named as HRV-1 (Han River Virome β-lactamase-1).


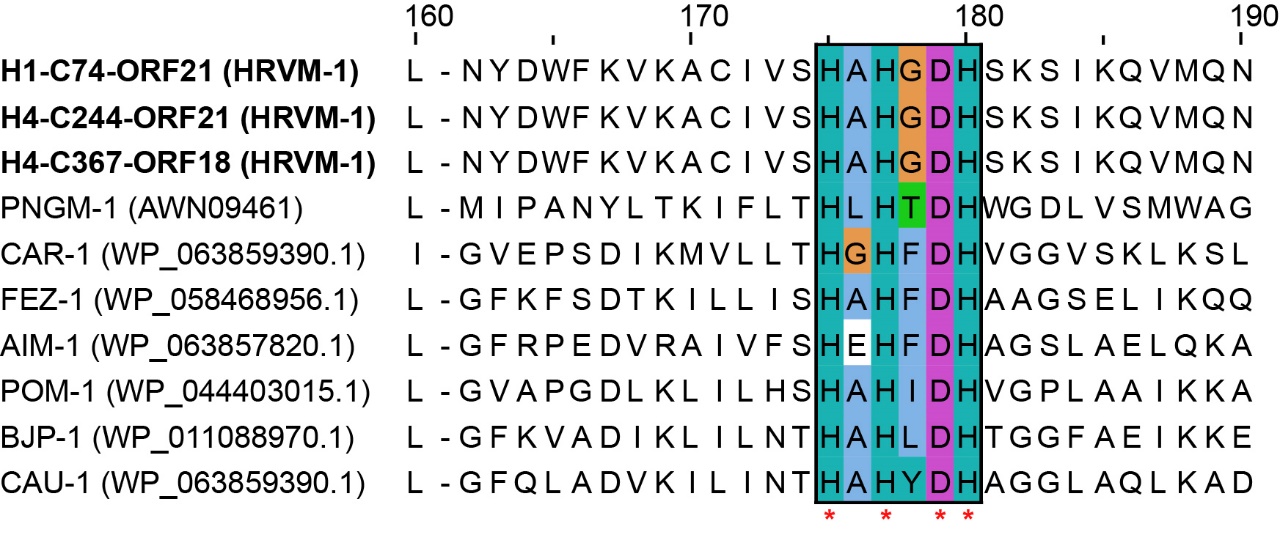


**Figure S11.** Amino acid sequence alignment of the three ORFs (H1-C74-ORF21, H4-C244-ORF21, and H4-C367-ORF18) with representative sequences of subclass B3 β-lactamases. The three ORFs retrieved from the Han River viromes contain conserved active sites of metallo-β-lactamase genes, which is “H-X-H-X-D-H.” The three ORFs show 100% sequence similarity with each other and were named as HRVM-1 (Han River Virome Metallo-β-lactamase-1).


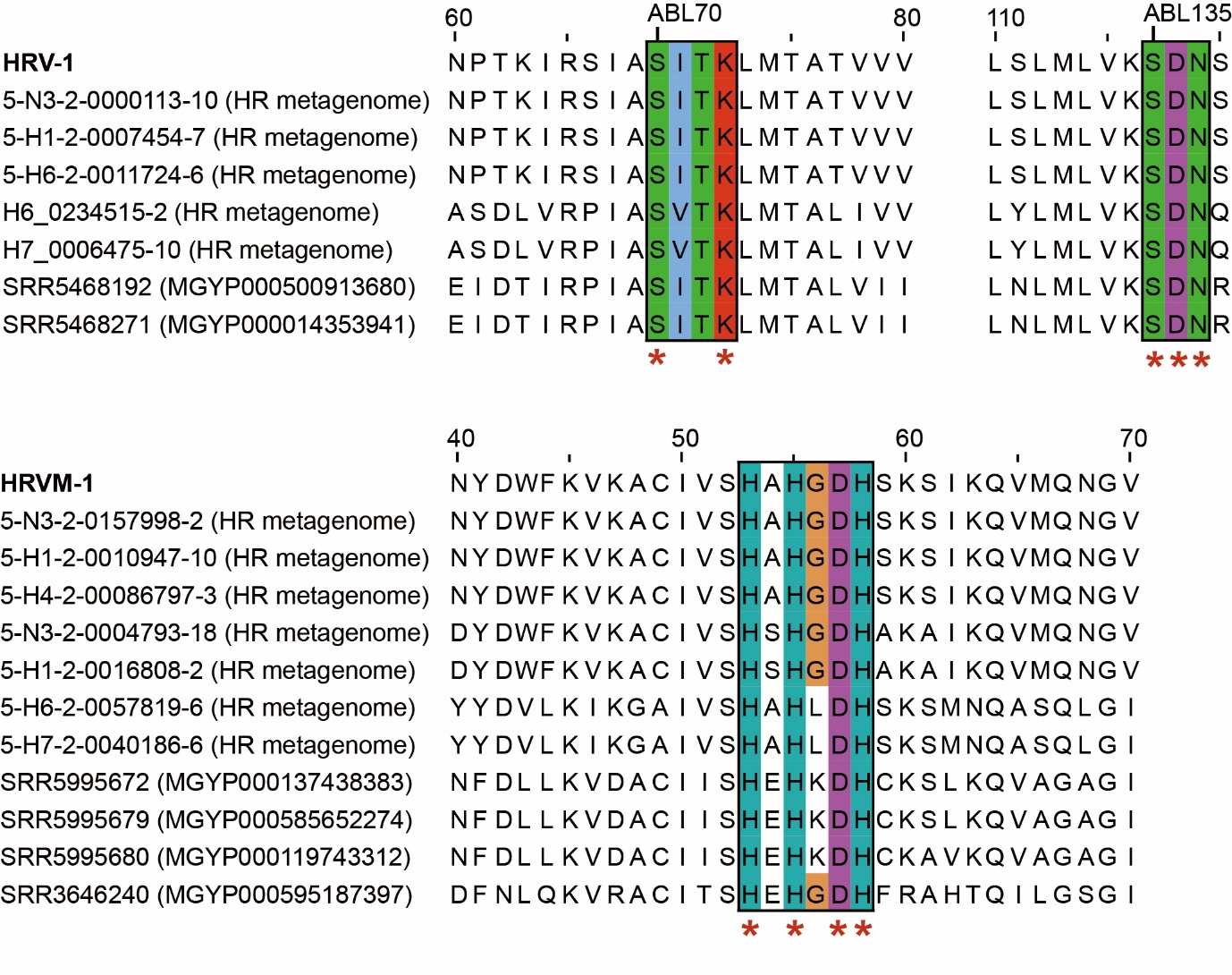


**Figure S12.** Amino acid sequence alignments of HRV-1 and HRVM-1 with highly related sequences discovered from the bacterial and viral metagenomes. The sequence names with sampling sites (N3, H1, H4, H6, and H7) indicate that the sequences were recovered from the Han River bacterial metagenomes that were concurrently prepared with the viral metagenome. The other metagenome sequences were retrieved from the EBI metagenome website (www.ebi.ac.uk/metagenomics/). SRR5468192 and SRR5468271 are bacterial metagenome sequences prepared from Colombia River estuary in the U.S. The viral metagenomes SRR5995672, 5995679, and 5995680 were collected from Singapore while the metagenome SRR3646240 was prepared from a clinical sample.


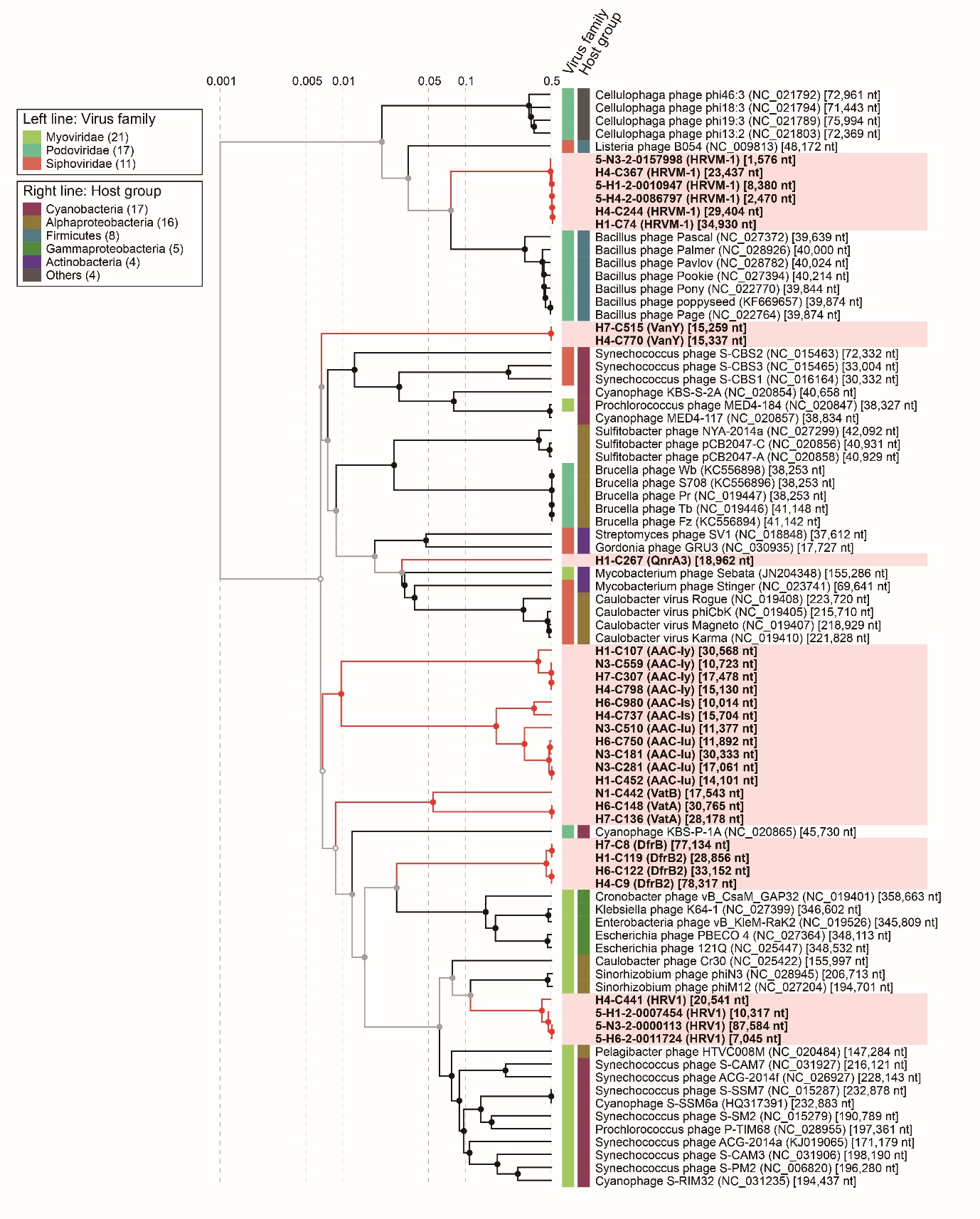


**Figure S13.** Viral proteomic tree of Han River viral contigs harboring ARG sequences constructed with the ViPTree program. The genome-wide proteomic tree for the ARG-carrying viral contig sequences were constructed based on the GenomeNet/Virus-Host database provided in the <http://www.genomie/jp/viptree>. The reference sequences used for the tree construction were manually selected based on the genome-wide tBLASTx scores.


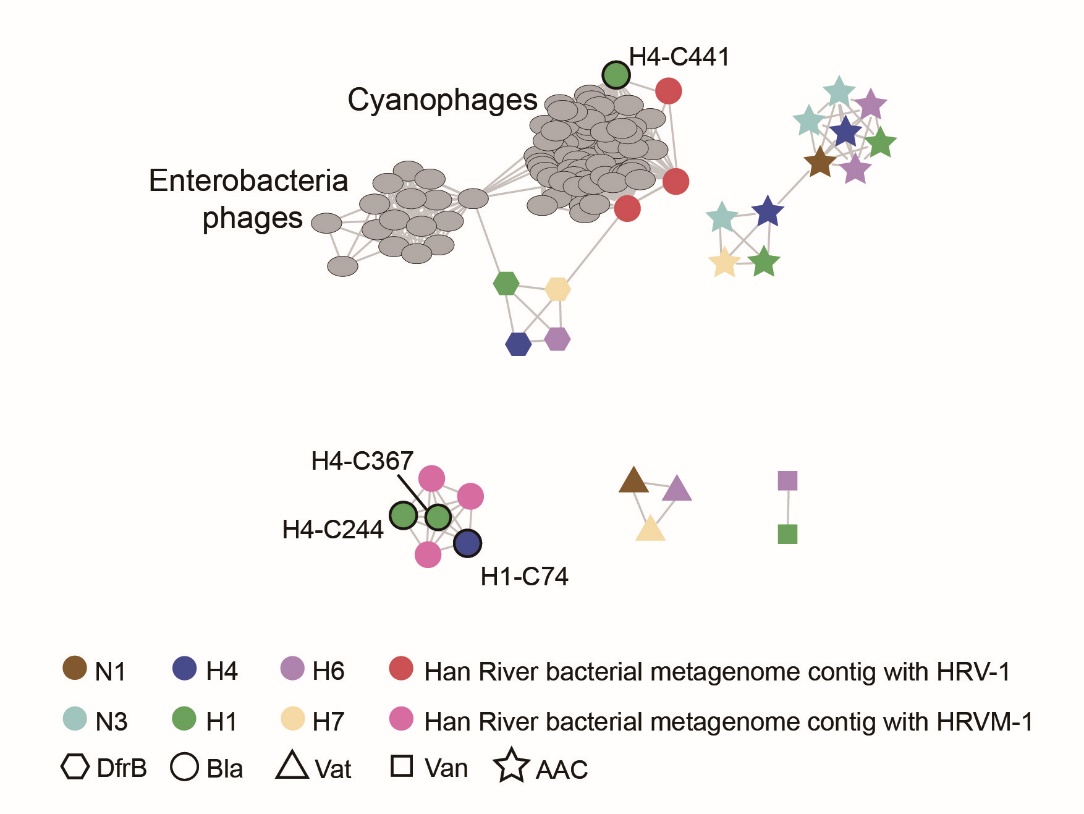


**Figure S14.** Gene-sharing network of Han River viral contigs harboring ARG sequences constructed with the vConTACT v.2.0 program. The network was constructed using the NCBI RefSeq database (release 95; downloaded on August 2019). The colors represent different sampling sites while the shape of the nodes indicate ARGs carried by the viral contigs.
